# Body height, body composition, and lifestyle of Czech high school students: implications for the most appropriate strategies supporting physical growth and preventing obesity

**DOI:** 10.1186/s40101-025-00418-2

**Published:** 2026-03-09

**Authors:** Pavel Grasgruber, Eduard Hrazdíra, Zuzana Hlavoňová, Nikola Stračárová, Dominik Bokůvka, Jan Cacek, Jan Huleš, Tomáš Kalina, Tereza Králová, Oldřich Racek, Lenka Svobodová, Marie Svobodová

**Affiliations:** 1 Faculty of Sports Studies, Masaryk University, Kamenice 5, Brno, 625 00 Czech Republic; 2 Centre of Sports Activities, Brno University of Technology, Brno, Czech Republic

**Keywords:** Height, Obesity, Nutrition, Dairy consumption, School catering, Parental education, Physical activity

## Abstract

**Background:**

This study summarizes anthropometric research that was conducted in two phases between 2015 and 2023. The aim of the first phase (2015–2016) was to map the current status of the height trend among adult high school students in the South Moravian region (the Brno-City District). The aim of the second phase (2016–2023) was to map the status of the height trend in three neighboring regions (Olomouc, Vysočina, Zlín) and to examine the relationship between body height, body composition, and some aspects of the students’ lifestyle.

**Methods:**

The study population consisted of 4655 individuals (2349 males, 2306 females) aged 18–22 from 71 high schools with a broad educational spectrum. The second phase of the research included 2045 individuals (1011 males, 1034 females) who completed questionnaires with questions about their lifestyle.

**Results:**

Based on the measurements of body height in a sample of evenly stratified categories of high schools in the Brno-City District, we arrived at an average of 180.5 ± 6.8 cm in males (*n* = 1338) and 166.5 ± 6.3 cm in females (*n* = 1272) aged 18–20 years. The planned measurements of body height in the three remaining regions were not completed due to the COVID epidemic, but the total averages remained practically unchanged (180.3 ± 6.8 cm in males, *n* = 2326; 166.5 ± 6.4 cm in females, *n* = 2299). An analysis using linear mixed-effect models showed that body height was best predicted by dairy consumption in males and by school lunch attendance in females. Low % body fat was most strongly associated with physical activity, distantly followed by high parental education and daily school lunch attendance. In both sexes, height was inversely related to % body fat, but this relationship was not mediated by physical activity.

**Conclusions:**

Taking into account other available data, it can be concluded that the secular trend of body height in the Czech Republic probably continues very slowly in males but stagnates in females. The inverse relationship between height and % body fat has an analogy in many other developed countries and suggests that a diet based on high-quality animal proteins should be the most effective strategy for supporting optimal physical development and preventing child obesity.

**Supplementary Information:**

The online version contains supplementary material available at https://doi.org/10.1186/s40101-025-00418-2.

## Introduction

Similar to some other European countries, the Czech Republic has had a strong tradition of nationwide anthropological surveys (*Celostátní antropologický výzkum, CAV*), which examined the current health status of the young population by collecting data on body height, body weight, and body diameters/circumferences. These health surveys have been conducted since 1951 at 10-year intervals, but the last of them took place in 2001. The seventh CAV in 2011 was not implemented because it did not receive a financial grant during the fading economic crisis and due to a lack of interest from ministerial institutions, the future prospects still remain unclear. For this reason, a large gap has arisen in the knowledge of the health status of Czech children and youth, precisely at a time when the risks of an increase in obesity are emphasized.

According to the last 6th CAV 2001, the young population of the Czech Republic reached a mean height of 180.2 cm in males (*n* = 1193) and 167.3 cm in females (*n* = 1701) at the age of 18 years [1], which are remarkably high values that indicate a very good state of nutrition. In fact, contemporary data show that the Czech youth were the tallest in Central Europe (even taller than their peers in Austria, Germany, and Switzerland) and among the tallest in the world [2]. At the same time, around 1870, a Czech military recruit in the Austro-Hungarian monarchy was only ~ 165 cm tall [3]. A positive height trend in the Czech population has been evident since the late 1880s and continued virtually uninterrupted throughout the twentieth century [4]. The greatest gains were recorded between 1961 and 1981 (~ 2 cm in males) and were followed by a deceleration between 1981 and 2001 (~ 1 cm in males) (Supplementary Fig. S1).

Currently, only regional data on body height are available, which are, however, very limited and do not allow a clear verdict on the state of the secular trend. In addition, it is necessary to take into account possible inter-regional differences in height. For instance, Jirkovský [5] reported a mean height of 179.5 cm based on a sample of 430 recruits aged 18–19 years from three Bohemian regions—Karlovy Vary, Plzeň, and Ústecký—measured between 2000 and 2001. The mean results varied between regions, with Karlovy Vary showing an average of 178.4 cm and Plzeň 180.7 cm, a difference of 2.3 cm. Despite the lack of nationally representative data, some surveys suggest that the positive trend in height may have continued. For example, Kopecký et al. [6] targeted adults aged 19 years and older, comprising students from Palacký University of Olomouc and attendees of public events between 2013 and 2015. Their findings indicated an average height of 179.9 cm for men (*n* = 479) and 166.9 cm for women (*n* = 1368) in the 19–29 age group, slightly surpassing those aged 30–44 years, who measured an average of 179.6 cm (*n* = 154) for men and 166.7 cm (*n* = 504) for women. Furthermore, another study by Kopecký et al. [7] assessed adolescents in the Olomouc and Moravskoslezský regions, revealing heights greater than those reported in CAV 2001; however, their sample sizes were quite limited and raise concerns about the representativeness of these findings.

In our own survey “Physical Fitness in the Czech Republic,” which took place between 2011 and 2013, we measured height and body composition in a sample of 1645 volunteers aged 18 + years [8]. This sample clearly included a tall, preselected population, which mostly consisted of participants in public sports events in Moravian regions, but it was interesting to observe persistent positive tendencies *within* the sample of men and the beginning of stagnation in women (Supplementary Fig. S2).

The longest records concerning the current development of obesity in Czech children and youth come from the survey *Zdraví dětí (*The Health of Children), which has been carried out by the National Institute of Public Health (Státní zdravotní ústav) since 1996 and examines age categories 5–17 years [9]. The survey indicates a gradual rise in obesity prevalence until 2011, followed by a stabilization in 2016. Specifically, obesity rates peak during early puberty at age 13 and subsequently decline due to rapid physical growth. A notable spike in child obesity was recorded in a 2021 survey, which must be attributed to the prolonged school lockdown during the COVID-19 pandemic (2020–2022) (Supplementary Fig. S3). For a more accurate assessment of the obesity trend, new research will be required after several years. Another longitudinal study, *Zdravá generace *(Health Generation), which analyzed data from 2002 to 2018 for children aged 11–15 years, reported a stagnation in obesity rates between 2010 and 2014, followed by a notable increase in 2018 [10]. The findings of this study indicate that obesity prevalence is closely related to social status, with children from lower-income families exhibiting the highest rates of obesity.

It is worth noting that in both of the aforementioned nationwide studies, it was observed that obesity rates were higher among boys compared to girls. This discrepancy prompts questions regarding the lifestyle choices of girls, suggesting they may be more health-conscious, or it could indicate a methodological flaw where obese girls opt not to participate in the measurements, leading to an underestimation of obesity rates in girls. In general, however, data from the OECD library [11] show that Czech children aged 5–9 years were below the average of 38 OECD countries in 2016.

Since representative data on body height have been missing for two decades, our initial aim was primarily to conduct an anthropometric survey of body height. This research (the first phase) started in June 2015 and incorporated adult high school students aged 18 + years, in whom we can assume that physical growth has been completed. Due to limited funding and human capacities, these measurements took place only in Brno (the center of the South Moravian region), but they allowed us to obtain at least provisional data with which we could work in our own studies.

Because during this survey, we noted significant differences in height and body composition across different types of schools, with students from vocational schools being significantly shorter and noticeably more overweight, we decided to launch a second phase in September 2016, the aim of which was to measure both body height and body composition using bioelectrical impedance on the InBody720 device. Thanks to better funding, we were able to include even three other neighboring regions (Vysočina, Olomouc, Zlín) and thus obtain a larger sample.

The second phase of the research would, therefore, have two main research objectives: first, we would have height data available from a total of four regions, which would significantly expand the geographical coverage of our study. Second, we would examine the roots of differences in height and body composition among different types of schools, which must be related to social background and differences in lifestyle.

For this purpose, we developed a questionnaire with basic questions regarding place of residence, parental education, diet, physical activity, and previous illnesses/injuries. Some of these important questions (such as the biological quality of the everyday diet) are not sufficiently addressed in the research on child obesity, although they could bring important insights into its causes. In the case of the Czech Republic, the role of school catering is particularly interesting. Founded in 1953, this system is considered one of the most sophisticated of its kind in the world, with a detailed breakdown of nutritional doses for each age category [12], and our initial hypothesis was that it contributes to the remarkable tallness of the Czech population. However, attempts are currently underway to reform this system, with greater emphasis on “healthy” plant-based ingredients and “unhealthy” animal ingredients (primarily in the form of red meat) [13]. An important secondary objective of our study was therefore to determine how school meals affect height and obesity, and whether the planned reform of school catering can really be beneficial in this regard.

## Methods

### The first phase of the survey (2015–2016): body height

The first phase of the research took place between June 2015 and May 2016 in the Brno-City District, the center of the South Moravian (Jihomoravský) region. This district included 58 high schools at that time, which were divided into four categories: A: elite schools *(gymnázium)* (*n* = 19), B: high schools with matriculation (*n* = 17), C1: vocational schools with a predominance of matriculation fields, or a balanced proportion of matriculation and non-matriculation fields (*n* = 7), C2: vocational schools with a predominance of non-matriculation fields (*n* = 6). This division was necessary because the students of these schools came from very different social groups, with potentially different lifestyles and varying dietary protein quality, and it was important to maintain a balanced proportion of these schools in the sample so that overrepresentation of one type of school could not distort the final results. Two art conservatories were not considered because they could not be classified into these categories, and we also left out seven schools for the mentally or physically disabled. Eventually, we measured 31 out of these 58 schools (53.4%). The sample included students from 3rd grade (mostly aged 17–18 years) and 4th grade (mostly aged 18–19 years), but only those aged at least 18 years and residing in the Czech Republic were included in the study. Body height was measured on the Seca portable stadiometer. In addition, the students filled out a short anonymous questionnaire with information about their age, place of residence, and the education of their parents.

Students of non-European origin living in the Czech Republic and Czech students of Romani origin were assessed separately from the main sample. Consequently, we created two special categories for East Asian (mostly Vietnamese) and Romani individuals. The separation of these two ethnic minorities is justified because data from Germany show that young Vietnamese are about one standard deviation (~ 5 cm) shorter than 17-year-old Germans [14]. Similarly, Roma women in the Czech Republic are approximately one standard deviation shorter than Czech women [15].

### The second phase of the survey (2016–2023): body height, body composition, and lifestyle

**Fig. 1:**
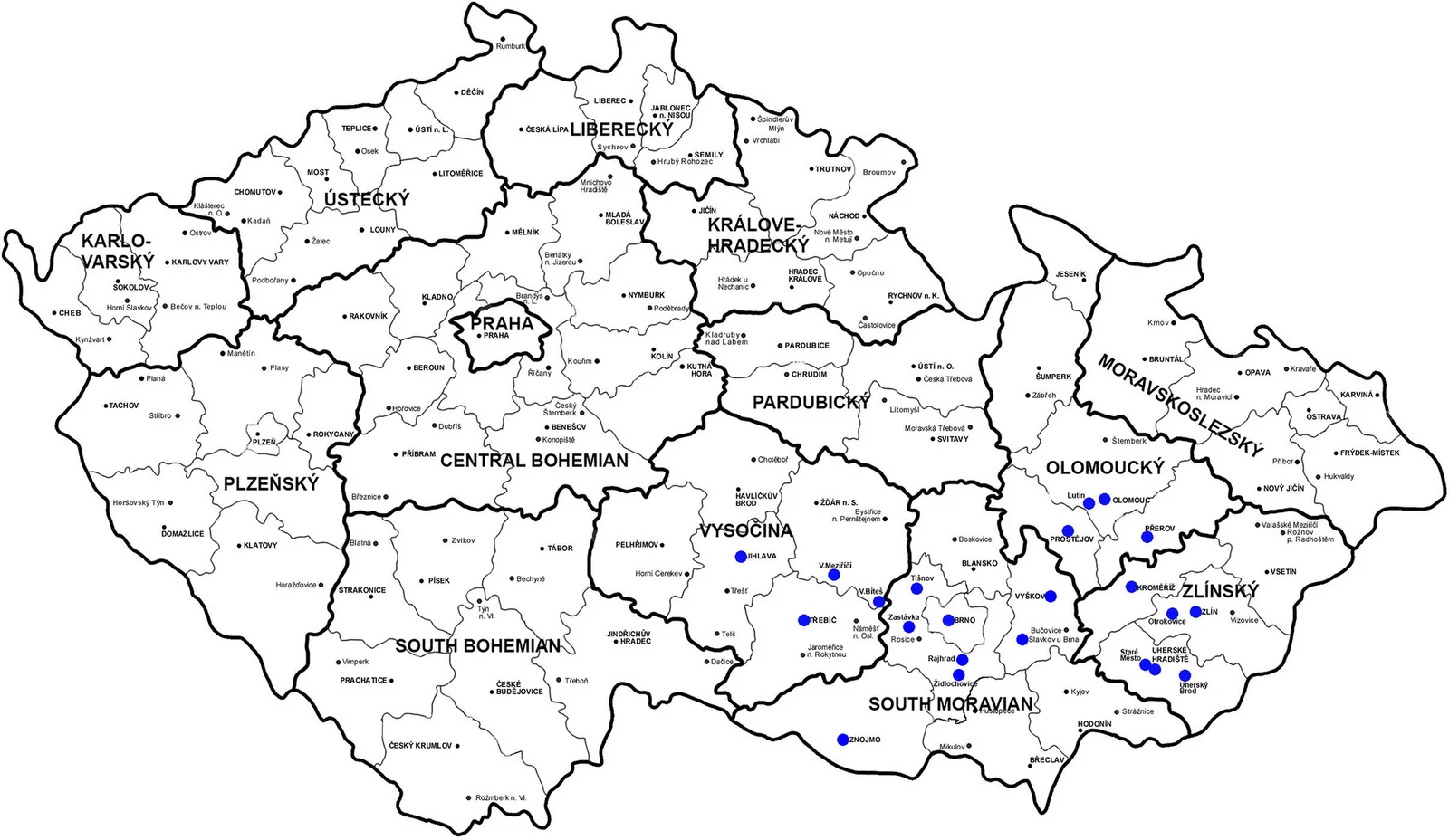
Map showing the locations where the research took place

The second phase started in September 2016, and 86% of the measurements were completed by December 2017. Several more schools were added in 2018–2019. The planned replenishment of samples in 2020 could no longer be implemented due to the outbreak of the COVID epidemic. Two schools were measured in May 2023, but due to the fact that students gained weight during the long COVID lockdown and did not attend school lunches, there was a legitimate concern that the relationships between lifestyle and body characteristics might be distorted, and the research was prematurely terminated. Altogether, 40 high schools with a wide educational spectrum were visited in four regions: South Moravian (Jihomoravský), Vysočina, Olomouc (Olomoucký), and Zlín (Zlínský) (Fig. 1). The schools in the regions of Vysočina, Olomouc, and Zlín were again divided according to their educational status.

Similar to the first phase, we focused on adult students aged at least 18 years and residing in the Czech Republic, with ethnic minorities of non-European origin being considered separately. The students first filled out a short questionnaire that included the most important questions about their age and environmental factors with demonstrable causal relationships to height and obesity (place of residence, parental education, diet, physical activity). These questions were designed to allow for reasonably reliable responses (Table 1). The students usually had 2–3 options to choose from. The dietary questions concerned the consumption of dairy products and the attendance of school lunches. Since dairy products contain proteins of the highest quality and are by far the strongest correlates of physical growth in the ecological comparison of 136 countries [2], the truthfulness of the answers could be verified by comparison with the values of body height. Understandably, it would not be realistic to expect students to indicate exactly how many years they have consumed dairy products daily, occasionally, and never, and we must assume that their answers reflect their predominant dietary patterns throughout their lives. Questions about the consumption of other foods did not seem meaningful because they could not be verified in a similar way and in many cases (e.g., distinguishing between the consumption of different types of meat), it would not be realistically possible.

**Table 1:** Questions related to lifestyle included in the questionnaire

Questions	Responses		
1. Do your parents have a college education?	Father (yes/no)	Mother (yes/no)	
2. Do you consume dairy products?	Daily	Sometimes	Never
3. Do you attend school lunches in high school?	Daily	Sometimes	Never
4. Did you attend school lunches in primary school?	Daily	Sometimes	Never
5. What is your level of physical activity?	Extreme	Moderate	Low
6. Are you a vegan or a vegetarian?	Yes	No	(If yes, how long?)
7. Have you been treated for a chronic disease during your life?	Yes	No	
8. Have you suffered a serious injury in your life?	Yes	No	

After completing the questionnaire, the students’ body height was measured on the Seca portable stadiometer and finally, they took body composition measurements on the InBody720 device. This device uses bioelectrical impedance and measures the resistance that the body exerts to weak electrical current. Because various body components have different electrical resistance, it is possible to estimate their mutual proportion, regardless of sex or ethnic origin. Compared to dual-energy X-ray absorptiometry (DXA), InBody devices somewhat underestimate body fat (by ~ 3%) and overestimate fat-free mass. However, given that DXA also has its limitations and probably overestimates true body fat, InBody720 can be regarded as one of the most reliable portable devices that are currently available [16]. A small subsample of students (348 males, 215 females) also underwent measurements of body proportions (arm span, sitting height) on a specially constructed device.

Although we did not keep any statistics on how many students (especially obese ones) deliberately avoided the measurements, from our cursory observations, it was a negligible percentage (~ 1–2%). On the contrary, the students showed great interest in the body composition measurement on the InBody720 device, and if some of them did not participate in the study, it was mainly due to their impatience, as this procedure was relatively time-consuming. For that reason, it can be assumed that the obtained data provide a very accurate picture of the current state of obesity in young Czech adults.

### Statistical analyses

For the statistical analyses of the obtained data, the statistical programs Statistica 14 and SPSS were used. The first part of the survey collected data on body height in the Brno-City District, which were first analyzed for normality. The presence of statistical differences among different types of schools was assessed using the ANOVA test. The only environmental factors collected during this survey, which could be used as independent variables for predicting height, were place of residence and parental education. Their potential relationship to height was assessed using the ANOVA test and linear mixed-effects models.

Data from the second part of the survey consisted of body height and body composition measurements. Height data were first used to assess the current status of the height trend in three regions (Olomouc, Vysočina, Zlín). Similar to the first phase of the survey, the regional averages of body height were analyzed for normality, and statistical differences among different types of schools were examined using the ANOVA test.

Subsequently, height and % body fat were selected as dependent variables in the analysis of the influence of lifestyle on physical characteristics. As independent variables, we used the place of residence, parental education, dairy consumption, school lunch attendance, and physical activity. The variable “school lunch attendance” was divided into two samples: a “high school only” sample, which tracked only school lunch attendance during 3–4 years of high school, and a smaller “primary and high school” sample covering a total of 12–13 school years, in which only about half of the students maintained stable eating habits.

According to the responses obtained from the questionnaires, each of these five independent variables was divided into 3–4 subgroups, among which statistical differences were assessed by the ANOVA test. Each subgroup was additionally divided into quintiles of body height and % body fat distribution, and trends across these quintiles were measured using *p*-values from Pearson linear correlations. The absolute contribution of independent variables to the values of body height and % body fat was estimated using linear mixed-effect models, which were preferred as the most relevant measure of statistical significance because the role of all examined independent variables is adjusted for each other. In these models, however, physical activity was a meaningful causal factor only in the analysis of % body fat.

## Results

### First phase of the survey

#### Body height

Information on body height was collected in 31 schools of the Brno-City District and was available for a total of 2610 individuals (1338 males, 1272 females) aged 18–20 years. These data are analyzed in detail in Table 2, and are broken down by age and school type. The averages of individual schools are displayed in Supplementary Table S1. In terms of normality of distribution, all five age groups of males in Table 2A were normally distributed according to the Kolmogorov–Smirnov test (*p* > 0.20), but not according to the Lilliefors test and the Shapiro–Wilk test. The Lilliefors test revealed a non-normal distribution (*p* < 0.05) in the age categories of 20 years, 18–19 years, and 18–20 years, and the Shapiro–Wilk test detected deviations from the Gaussian curve in the age categories of 18 years, 18–19 years, and 18–20 years. In general, the male samples were characterized by a tendency towards positive skewness (a disproportionate number of very tall males) and higher kurtosis (more outliers). However, given the large sample size and the balanced stratification of the four school types, it is unlikely that the composition of our samples was distorted, and the seeming irregularity of data distribution can be explained by the high sensitivity of the normality tests used, in particular the Shapiro–Wilk test [17]. In contrast, all the age groups of females were normally distributed according to all three tests (*p* > 0.10).

**Table 2:** **A**. Average height of males in the Brno-City district measured during the first phase of the survey, by age and type of school. **B**. Average height of females in the Brno-City district measured during the first phase of the survey, by age and type of school

**A (Males)**											
**Age **	**A-schools (n=11)**		**B-schools (n=11)**		**C1-schools (n=4)**		**C2-schools (n=4)**		**ANOVA**	**All schools (n=30)**	
	*n*	Height	*n*	Height	*n*	Height	n	Height		*n*	Height
18	*285*	181.3±6.4	*251*	180.7±7.1	*116*	180.7±7.3	*122*	179.3±6.6	*0.062*	*774*	180.7±6.8
19	*162*	180.9±6.0	*157*	180.1±7.0	*77*	180.9±6.6	*68*	179.2±7.5	*0.243*	*464*	180.4±6.7
20	*15*	180.7±7.1	*33*	178.4±5.6	*30*	180.4±7.7	*22*	178.6±5.1	*0.489*	*100*	179.4±6.4
18-19	***447***	**181.1±6.3**	***408***	**180.5±7.0**	***193***	**180.8±7.1**	***190***	**179.2±6.9**	***0.012***	*1238*	180.6±6.8
18-20	***462***	**181.1±6.3**	***441***	**180.3±7.0**	***223***	**180.8±7.1**	***212***	**179.2±6.8**	***0.005***	*1338*	180.5±6.8

**B (Females)**											
**Age**	**A-schools (n=11)**		**B-schools (n=11)**		**C1-schools (n=4)**		**C2-schools (n=3)**		**ANOVA**	**All schools (n=29)**	
	*n*	Height	*n*	Height	*n*	Height	*n*	Height		*n*	Height
18	***285***	**167.7±6.2**	***332***	**165.7±6.1**	***70***	**165.7±5.9**	***43***	**165.5±6.0**	***<0.001***	*730*	166.5±6.2
19	***180***	**167.2±6.2**	***203***	**166.0±6.4**	***60***	**167.7±6.4**	***38***	**164.2±6.7**	***0.012***	*481*	166.5±6.4
20	*5*	168.0±7.7	*20*	166.1±6.2	*19*	168.5±7.8	*17*	165.4±6.5	*0.532*	*61*	166.8±6.9
18-19	***465***	**167.5±6.2**	***535***	**165.8±6.2**	***130***	**166.6±6.2**	***81***	**164.9±6.4**	***<0.001***	*1211*	166.5±6.2
18-20	***470***	**167.5±6.2**	***555***	**165.8±6.2**	***149***	**166.9±6.4**	***98***	**164.9±6.4**	***<0.001***	*1272*	166.5±6.3

Significant intergroup differences are highlighted in bold

At first glance, there were significant differences across various types of schools: males and females from the elite A-schools were the tallest, those from the B and C1 schools were of moderate height, and those from the C2 vocational schools were by far the shortest. In the total sample of 18–20-year-olds, the difference between the A and C2 schools reached 1.9 cm in males and 2.6 cm in females, and was highly significant. The A-schools had the greatest proportion of students whose both parents had a college diploma (40.3% in males, 38.7% in females). In the B-schools (9.8% in males, 7.6% in females), the C1-schools (12.6% in males, 2.7% in females), and the C2-schools (6.6% in males, 10.2% in females), this percentage decreased dramatically.

Compared to 17–18-year-olds in the 6th CAV [1], the overall average for males (180.5 cm) is slightly higher, but the average for females (166.5 cm) is lower. The difference between males and females (14 cm) is thus 1 cm higher than in previous CAV surveys. It was the lowest at the elite A-schools (13.6 cm), but > 14 cm at other types of schools. These unexpected findings emerged at the very beginning of the research and did not disappear, despite our best efforts to balance the ratio of individual types of schools and increase the sample size.

#### Body height vs. place of residence and parental education

Information on parental education and place of residence were the only environmental predictors of height collected during the first phase of the survey. The place of residence was known in 2604 individuals (1334 males and 1270 females). Although residents from big cities (in this case, almost exclusively the city of Brno with ~ 400,000 inhabitants) were always the tallest, the differences compared to residents from villages and smaller towns were small and insignificant (Table 3A,B). In other words, the availability of quality nutrition and health care appears to be quite good in settlements of any size. Parental education was reported by 2610 individuals (1338 males and 1272 females) and shows a significant effect of college education on height, especially in females (Table 3). These results were essentially confirmed by a linear mixed-effect model, in which the place of residence played a marginal role, but having both parents with college education added 1.21 cm in males and 1.63 cm in females.

**Table 3:** **A**. Average height of males aged 18-20 years measured during the first phase of the survey, by place of residence and parental education. **B**. Average height of females aged 18-20 years measured during the first phase of the survey, by place of residence and parental education

**A (Males)**								
**Place of residence (n=1334)**			**Parental education (college) (n=1338)**			**Linear mixed-effect model (Intercept: 180.09 cm)**		
Inhabitants (n)	*n*	Height	Parents	*n*	Height (cm)	Subcategories	Estimate	*p*-value
>100,000	761	180.6±6.8	Both	*271*	**181.3±6.4**	>100,000	*0.00	
5000-100,000	156	180.2±6.9	Father only	*186*	**180.7±7.1**	5000-100,000	-0.24	*0.68*
0-5000	417	180.3±6.7	Mother only	*132*	**181.0±6.5**	0-5000	-0.05	*0.91*
			None	*749*	**180.0±6.8**			
						**Both**	**1.21**	***0.013***
						Father only	0.69	*0.21*
						Mother only	0.93	*0.15*
*ANOVA*		*0.673*	*ANOVA*		***0.048***	None	*0.00	

**B (Females)**								
**Place of residence (n=1270)**			**Parental education (college) (n=1272)**			**Linear mixed-effect model (Intercept: 166.18 cm)**		
Inhabitants (n)	*n*	Height	Parents	*n*	Height (cm)	Subcategories	Estimate	*p*-value
>100,000	624	166.8±6.3	Both	*238*	**167.7±5.9**	>100,000	*0.00	
5000-100,000	180	166.1±6.5	Father only	*166*	**166.7±6.0**	5000-100,000	-0.51	*0.34*
0-5000	466	166.3±6.2	Mother only	*124*	**167.0±5.9**	0-5000	-0.22	*0.59*
			None	*744*	**166.0±6.5**			
						**Both**	**1.63**	***0.001***
						Father only	0.61	*0.27*
						Mother only	1.00	*0.10*
*ANOVA*		*0.211*	*ANOVA*		***0.002***	None	*0.00	

Significant intergroup differences are highlighted in bold

*This parameter (intercept) represents a standard within each variable and is set to zero. The sum of values from individual subcategories can predict theoretical values of body height, relative to the intercept. For example, a female from a small town with 0-5000 inhabitants (-0.22 cm) and with both college-educated parents (+1.63 cm), would be 167.59 cm tall (166.18 – 0.22 + 1.63 = 167.59)

### Second phase of the survey

#### Body height

During the second phase of the survey, which took place in four regions, height was available in a sample of 2045 individuals (1011 males, 1034 females) aged 18–22 years. However, due to the premature termination of the research after the COVID epidemic, the B-schools and especially the C1-schools were underrepresented, and hence this sample cannot be regarded as sufficiently representative. Due to the small number of the C1 schools (*n* = 2), the categories C1 and C2 were merged together. Nevertheless, the average heights (180.1 ± 6.9 cm in men, 166.5 ± 6.4 cm in women) were almost identical to those in the first phase. The samples of males and females had a completely normal statistical distribution (*p* ≥ 0.15) according to all three normality tests (Kolmogorov–Smirnov, Lilliefors, Shapiro–Wilk) (Table 4). In contrast to the results from the first phase, males from the B-schools were unexpectedly taller (+ 1 cm) than males from the elite A-schools, but this discrepancy can be ascribed to the uneven representation of school types in individual regions. In any case, this difference was not significant, and the social background of the students was very similar to that in the first phase: the A-schools had the highest proportion of students who had both parents with college education (30.7% in males, 25.4% in females), whereas this percentage was much lower at the B-schools (5.0% in males, 3.2% in females) and the C-schools (4.8% in males, 2.5% in females). The averages of individual schools are displayed in Supplementary Table S2.

**Table 4:** Body height in males and females aged 18–22 years from four Czech regions measured during the second phase of the survey, by type of school

	A-schools (*n* = 18)		B-schools (*n* = 8)		C-schools (*n *= 14)		ANOVA	All schools (*n *= 40)	
	*n*	Height (cm)	*n*	Height (cm)	*n*	Height (cm)		*n*	Height (cm)
Males	*326*	179.9 ± 7.1	*229*	180.9 ± 6.9	*456*	179.8 ± 6.7	*0.104*	*1011*	180.1 ± 6.9
Females	***459***	**167.4 ± 6.1**	***216***	**165.6 ± 6.1**	***359***	**165.9 ± 6.9**	** < *0.001***	*1034*	166.5 ± 6.4

Note that due to the low representation of the C1 schools (*n* = 2), the C1 and C2 schools were merged together. Significant intergroup differences are highlighted in bold

When samples of 18–20-year-olds from both phases are combined, the average heights change only a little (180.3 ± 6.8 cm in males, *n* = 2326; 166.5 ± 6.4 cm in females, *n* = 2299). Regional samples sorted according to the place of residence have mostly suboptimal size, both at the level of major regions (Tables 5 and 6) and districts (Supplementary Table S3), but there is a fairly good correlation between male and female heights in 16 districts with at least 20 measured individuals (*r* = 0.47, *p* = 0.068), with only two notable outliers (Uherské Hradiště and Žďár nad Sázavou) (Supplementary Fig. S4). Also noteworthy is the high concordance between the averages from the districts of Brno-City (180.6 cm in males, 166.8 cm in females) and Brno-Country (180.6 cm in males, 166.6 cm in females), which makes it possible to create standards for future research.

**Table 5:** Body height in the total sample of males aged 18–20 years, by place of residence and type of school. Six regions with a small number of males (*n* = 21 in total) are not listed. Two males did not report their place of residence

Place of residence (regions)	A-schools (*n* = 29)		B-schools (*n* = 19)		C-schools (*n* = 22)		All schools (*n* = 70)		
	*n*	Height (cm)	*n*	Height (cm)	*n*	Height (cm)	*n*	Height (cm)	min–max
South Moravian	*608*	180.7 ± 6.5	*464*	180.5 ± 6.8	*518*	179.6 ± 6.9	*1590*	180.3 ± 6.7	159.6–205.1
1st phase	*457*	181.1 ± 6.3	*416*	180.4 ± 6.9	*416*	179.9 ± 6.9	*1289*	180.5 ± 6.7	
2nd phase	*151*	179.5 ± 6.9	*48*	181.3 ± 6.2	*102*	178.5 ± 6.5	*301*	179.5 ± 6.7	
Olomouc	*75*	180.9 ± 8.0	*135*	181.0 ± 7.2	*168*	180.5 ± 7.0	*378*	180.8 ± 7.3	157.5–202.4
Vysočina	*45*	179.6 ± 6.2	*17*	177.1 ± 6.0	*78*	181.1 ± 6.9	*140*	180.2 ± 6.7	165.7–194.5
Zlín	*59*	180.1 ± 6.9	*45*	179.9 ± 6.8	*91*	179.0 ± 6.0	*195*	179.5 ± 6.4	158.6–200.5
**Total sample**	***788***	**180.6 ± 6.7**	***668***	**180.5 ± 6.9**	***868***	**179.9 ± 6.9**	***2326***	**180.3 ± 6.8**	**157.5–205.1**

**Table 6:** Body height in the total sample of females aged 18–20 years, by type of school. Six regions with a small number of females (*n* = 42 in total) are not listed. One female did not report her place of residence

Place of residence (regions)	A-schools (*n* = 29)		B-schools (*n* = 19)		C-schools (*n* = 20)		All schools (*n* = 68)		
	*n*	Height (cm)	*n*	Height (cm)	*n*	Height (cm)	*n*	Height (cm)	
South Moravian	*656*	167.5 ± 6.1	*595*	165.6 ± 6.0	*401*	165.9 ± 6.7	*1652*	166.4 ± 6.2	146.3–186.8
1st phase	*463*	167.5 ± 6.2	*472*	165.7 ± 5.9	*228*	166.0 ± 6.4	*1163*	166.5 ± 6.2	
2nd phase	*193*	167.4 ± 5.9	*123*	165.5 ± 6.1	*173*	165.8 ± 7.0	*489*	166.4 ± 6.4	
Olomouc	*87*	167.6 ± 6.1	*20*	165.8 ± 7.4	*49*	164.9 ± 7.7	*156*	166.5 ± 6.9	147.8–185.4
Vysočina	*59*	167.8 ± 6.0	*58*	166.2 ± 7.1	*74*	167.1 ± 6.8	*191*	167.0 ± 6.6	153.1–183.5
Zlín	*121*	167.0 ± 6.7	*73*	166.7 ± 6.4	*63*	166.2 ± 6.1	*257*	166.7 ± 6.5	151.1–188.6
**Total sample**	***929***	**167.4 ± 6.2**	***770***	**165.8 ± 6.2**	***599***	**166.0 ± 6.7**	***2299***	**166.5 ± 6.4**	**146.3–188.6**

Visible ethnic minorities of non-European origin, which were assessed separately, consisted of Roma and East Asians (mainly Vietnamese). However, the Roma sample included only a few students, which was expected because it is generally known that this ethnic group does not pursue higher education. The East Asian sample was also small (11 males aged 19.0 ± 1.0 years, 171.5 ± 5.9 cm; 10 females aged 19.1 ± 1.1 years, 156.7 ± 5.2 cm), but it was markedly taller than Vietnamese men and women aged 22–26 years in the Vietnamese General Nutrition Survey 2009–2010 (164.4 cm and 153.4 cm, respectively) [2]. These numbers can also be compared to the height of young Vietnamese people in Germany: 173.1 ± 6.5 cm in males, 160.7 ± 6.0 cm in females [14].

#### Body composition

**Fig. 2:**
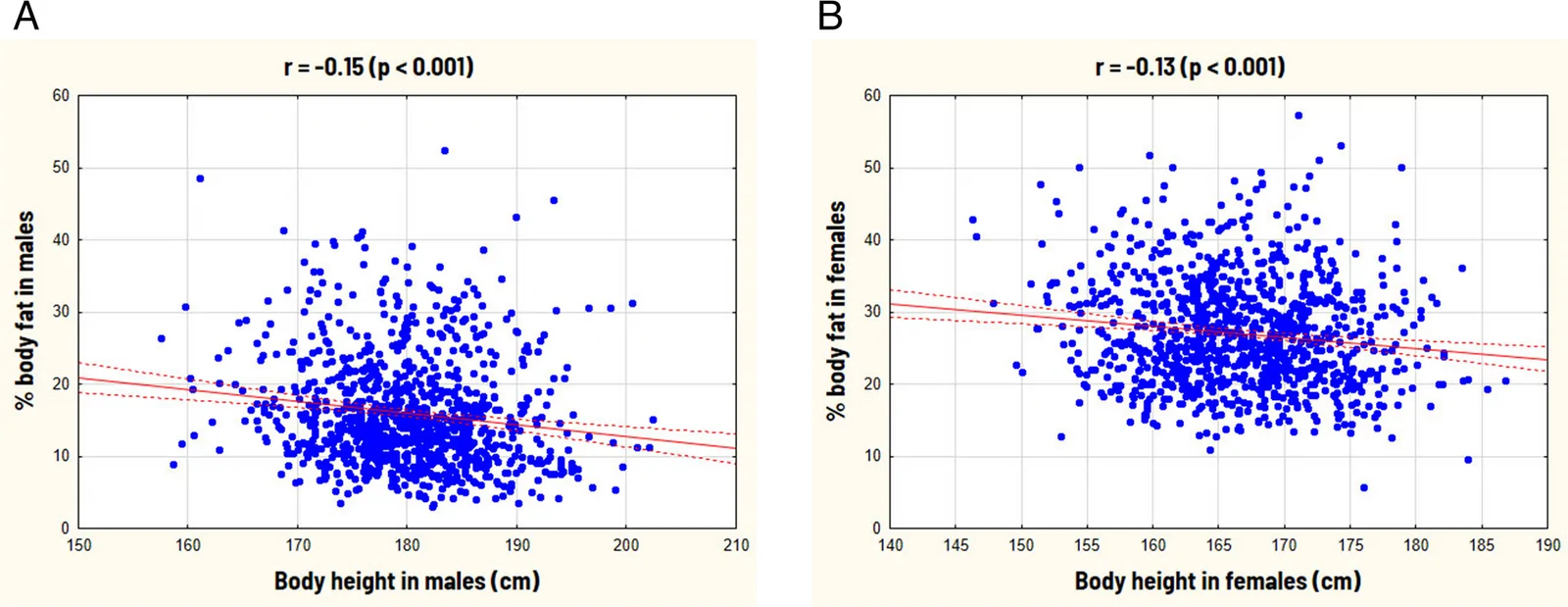
**A** Relationship between body height and % body fat in males from four Czech regions. **B** Relationship between body height and % body fat in females from four Czech regions

**Fig. 3:**
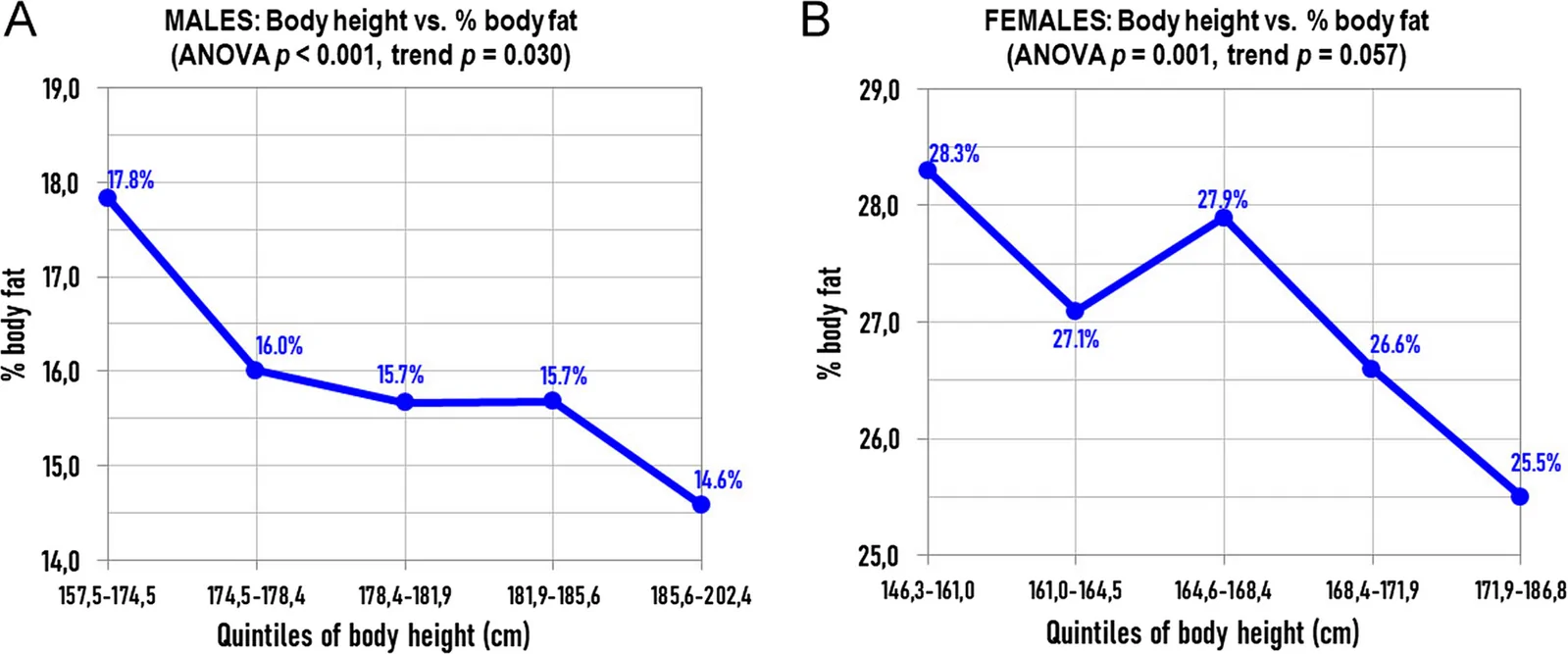
**A** Relationship between body height and % body fat in males from four Czech regions, by height quintile. **B** Relationship between body height and % body fat in females from four Czech regions, by height quintile

Out of 2045 individuals (1011 males, 1034 females) aged 18–22 years who underwent measurements of body height and filled out a questionnaire during the second phase of the survey, body composition measurements were available for 2030 individuals (1002 males and 1028 females) (Table 7). The analysis revealed notable differences in adiposity indicators, which were lowest among students in A-schools and highest in C-schools. Visually, the prevalence of obesity in C-school females was striking. Males from A-schools also showed a greater proportion of muscle mass, while the muscle mass index was highest among C-school males. Similar tendencies can be observed in females. This finding is not surprising, as the excessive weight of body fat stimulates the development of muscle mass, even independently of physical activity [18]. Another notable finding is that the relationship between height and % body fat is inverse and highly significant in both sexes (Fig. 2A, B). It is true that the *r*-values are very weak, but this is understandable because the range of individual values of % body fat is very wide, approximately 5–40% in males and 10–50% in females. When the average values of % body fat are calculated for each height quintile, the inverse trend is clearly visible, although it is significant only in males and approaches significance in females (Fig. 3A, B).

**Table 7:** Body composition in males and females from four Czech regions measured with the InBody720 device during the second phase of the survey, by type of school

**MALES**	**A-schools**	**B-schools**	**C-schools**	**ANOVA**	**All schools (min-max)**	
*n (males)*	*317*	*229*	*456*		*1002*	
Height (cm)	179.9 ±7.2	180.9 ±6.9	179.8 ±6.7	*0.100*	180.1 ±6.9	157.5-202.4
Weight (kg)	**74.2 ±11.8**	**77.6 ±15.9**	**79.0 ±14.2**	***<0.001***	77.2 ±14.0	50.7-159.9
Body mass index (kg/m^2^)	**22.93 ±3.36**	**23.69 ±4.49**	**24.42 ±4.04**	***<0.001***	23.78 ±4.00	15.81-42.79
Body fat (kg)	**10.9 ±6.9**	**13.4 ±10.3**	**14.3 ±8.9**	***<0.001***	13.1 ±8.8	2.0-72.8
% body fat	**14.13 ±6.63**	**16.03 ±8.31**	**17.19 ±7.66**	***<0.001***	15.95 ±7.62	3.00-52.52
% overweight (>20% BF)	14.8	25.3	30.7		24.5	
Visceral fat (cm^3^)	**59.4 ±29.0**	**70.7 ±40.6**	**77.4 ±41.0**	***<0.001***	70.2 ±38.3	5.0-486.3
Waist-to-hip ratio	**0.855 ±0.042**	**0.865 ±0.051**	**0.878 ±0.047**	***<0.001***	0.868 ±0.047	0.741-1.018
Muscle mass (kg)	36.00 ±4.80	36.49 ±5.01	36.76 ±4.87	*0.102*	36.46 ±4.89	22.88-57.76
Muscle mass index (kg/m^2^)	**11.10 ±1.16**	**11.13 ±1.23**	**11.35 ±1.21**	***0.007***	11.22 ±1.21	7.72-16.08
% muscle mass	**48.81 ±3.86**	**47.68 ±4.73**	**47.04 ±4.39**	***<0.001***	47.75 ±4.38	26.72-55.71
Bone mineral content (kg)	3.59 ±0.51	3.66 ±0.54	3.68 ±0.52	*0.059*	3.64 ±0.52	2.32-5.52

**FEMALES**	**A-schools**	**B-schools**	**C-schools**	**ANOVA**	**All schools (min-max)**	
*n (females)*	*453*	*216*	*359*		*1028*	
Height (cm)	**167.3 ±6.2**	**165.6 ±6.1**	**165.9 ±6.9**	***<0.001***	166.5 ±6.5	146.3-186.8
Weight (kg)	**60.7 ±9.9**	**61.5 ±12.7**	**63.4 ±13.2**	***0.005***	61.8 ±11.8	39.4-145.6
Body mass index (kg/m^2^)	**21.64 ±3.25**	**22.40 ±4.20**	**22.99 ±4.33**	***<0.001***	22.27 ±3.91	15.52-47.93
Body fat (kg)	**15.9 ±6.7**	**17.8 ±8.9**	**18.9 ±9.3**	***<0.001***	17.4 ±8.3	3.8-77.4
% body fat	**25.52 ±6.65**	**27.77 ±7.86**	**28.62 ±8.00**	***<0.001***	27.08 ±7.53	5.8-57.4
% overweight (>28% BF)	30.9	44.4	53.6		40.5	
Visceral fat (cm^3^)	**49.9 ±27.0**	**56.6 ±31.4**	**64.6±54.1**	***<0.001***	56.5 ±39.8	6.5-888.8
Waist-to-hip ratio	**0.831 ±0.047**	**0.840 ±0.053**	**0.852±0.056**	***<0.001***	0.840 ±0.052	0.720-1.044
Muscle mass (kg)	24.48 ±3.05	23.85 ±3.34	24.35±3.64	*0.071*	24.30 ±3.33	15.70-39.43
Muscle mass index (kg/m^2^)	8.72 ±0.80	8.67 ±0.88	8.82±0.98	*0.111*	8.75 ±0.88	6.38-12.62
% muscle mass	**40.74 ±3.75**	**39.38 ±4.31**	**39.02±4.45**	***<0.001***	39.85 ±4.20	23.67-54.28
Bone mineral content (kg)	2.66 ±0.64	2.61 ±0.36	2.66±0.37	*0.529*	2.65 ±0.51	1.65-4.24

Note that the waist–hip ratio was measured with InBody720 and not manually

Significant intergroup differences are highlighted in bold

According to the cut-off points defined by WHO [19], 29.6% of males and 18.8% of females would fall into the overweight category (BMI ≥ 25 kg/m^2^), and 7.4% of males and 5.1% of females would fall into the obese category (BMI ≥ 30 kg/m^2^). The manufacturer of InBody720 defines a normal % body fat range as 10–20% in males and 18–28% in females. From this view, 24.0% of males and 40.4% of females would be overweight. The disproportionate prevalence of excessive body fat in females is evident in the C-schools, where the average female had 28.5% body fat and was, therefore, overweight.

#### Body height vs. lifestyle

This analysis examined the relationship between body height and lifestyle factors in 2045 individuals (1011 males, 1034 females) aged 18–22 years who filled out a questionnaire during the second phase of the survey (Table 8, Supplementary Figs. S5 and S6). The first of these factors is place of residence, which showed no significant relationship to height either across subcategories of settlement size or across height quintiles. This replicates the result from the first phase of the research. Nevertheless, students from big cities were again the tallest. The importance of parental education was less pronounced than in the first phase of the survey. Statistical significance was observed only in females whose height in quintiles increased quite linearly with the rising proportion of college-educated fathers and especially both college-educated parents. The most likely explanation for the less significant results is a smaller sample size, as the observed tendencies otherwise remained similar.

**Table 8:** Average percentage of individuals with selected lifestyle characteristics in each quintile of body height

**MALES**	***n***	**Height (cm)**	**Quintiles of body height in males (cm)**					***p* for trend**
			**157.5-174.5**	**174.5-178.4**	**178.4-181.9**	**181.9-185.6**	**185.6-202.4**	
% body fat	*1002*		**17.83±8.01**	**16.01±6.94**	**15.66±7.07**	**15.68±7.83**	**14.58±7.89**	***0.030***
PLACE OF RESIDENCE	*1009*	180.1±6.9						
>100 000 inhabitants	*155*	181.1±6.3	7.9	18.8	19.2	12.9	18.0	*0.435*
5000-100 000 inhab.	*382*	180.0±7.0	39.1	38.6	32.0	41.1	38.5	*0.926*
0-5000 inhabitants	*472*	179.9±6.9	53.0	42.6	48.8	46.0	43.5	*0.306*
*ANOVA*		*0.158*	100% *(202)*	100% *(202)*	100% *(203)*	100% *(202)*	100% *(200)*	
PARENTAL EDUCATION (college)	*1006*	180.1±6.9						
Both parents	*132*	180.6±7.1	10.9	16.9	13.4	9.5	14.9	*0.960*
Father only	*119*	180.9±6.8	10.0	10.4	9.9	16.9	11.9	*0.335*
Mother only	*122*	180.6±6.7	9.0	13.9	10.9	14.9	11.9	*0.439*
No parent	*633*	179.8±6.8	70.1	58.7	65.8	58.7	61.2	*0.318*
*ANOVA*		*0.219*	100% *(201)*	100% *(201)*	100% *(202)*	100% *(201)*	100% *(201)*	
DAIRY CONSUMPTION	*1006*	180.1±6.9						
Daily	*471*	**181.1±7.1**	**39.0**	**40.8**	**45.5**	**50.5**	**58.2**	***0.004***
Sometimes	*527*	**179.2±6.5**	**59.5**	**59.2**	**53.5**	**49.0**	**40.8**	***0.008***
Never	*8*	**178.6±7.3**	1.5	0.0	1.0	0.5	1.0	*0.824*
*ANOVA*		***<0.001***	100% *(200)*	100% *(201)*	100% *(202)*	100% *(202)*	100% *(201)*	
SCHOOL LUNCHES^primary+high school^	*549*	180.0±6.7						
Daily	*409*	180.4±6.9	66.7	76.8	70.1	79.5	80.0	*0.119*
Sometimes	*72*	179.3±6.7	16.7	8.9	16.2	11.6	12.0	*0.599*
Never	*68*	178.5±5.8	**16.7**	**14.3**	**13.7**	**8.9**	**8.0**	***0.007***
*ANOVA*		*0.061*	100% *(108)*	100% *(112)*	100% *(117)*	100% *(112)*	100% *(100)*	
SCHOOL LUNCHES^high school only^	*1008*	180.1±6.9						
Daily	*471*	180.3±6.8	41.8	50.0	45.5	51.0	45.3	*0.583*
Sometimes	*238*	179.7±6.8	27.9	18.8	23.3	27.2	20.9	*0.720*
Never	*299*	180.2±7.0	30.3	31.2	31.2	21.8	33.8	*0.893*
*ANOVA*		*0.525*	100% *(201)*	100% *(202)*	100% *(202)*	100% *(202)*	100% *(201)*	
PHYSICAL ACTIVITY	*991*	180.1±6.9						
Extreme	*122*	180.6±6.0	9.2	14.5	10.6	14.6	12.6	*0.441*
Moderate	*636*	180.3±7.1	66.3	58.5	67.2	61.6	67.3	*0.740*
Low	*233*	179.5±6.7	24.5	27.0	22.2	23.7	20.1	*0.153*
* ANOVA*		*0.264*	100% *(196)*	100% *(200)*	100% *(198)*	100% *(198)*	100% *(199)*	

**FEMALES**	***n***	**Height (cm)**	**Quintiles of body height in females (cm)**					***p* for trend**
			**146.3-161.0**	**161.0-164.5**	**164.6-168.4**	**168.4-171.9**	**171.9-186.8**	
% body fat	*1028*		28.31±7.90	27.06±6.91	27.91±7.86	26.56±6.98	25.54±7.67	*0.057*
PLACE OF RESIDENCE	*1031*	166.5±6.4						
>100 000 inhabitants	*92*	167.0±6.6	9.7	5.3	10.2	9.7	9.7	*0.586*
5000-100 000 inhab.	*448*	166.7±6.7	42.7	41.3	37.6	49.3	46.4	*0.354*
0-5000 inhabitants	*491*	166.2±6.3	47.6	53.4	52.2	41.1	44.0	*0.297*
*ANOVA*		*0.311*	100% *(206)*	100% *(206)*	100% *(205)*	100% *(207)*	100% *(207)*	
PARENTAL EDUCATION (college)	*1032*	166.5±6.4						
Both parents	*132*	**167.6±6.3**	**9.2**	**11.1**	**13.6**	**15.0**	**15.0**	***0.010***
Father only	*118*	**167.2±6.2**	**8.7**	**11.1**	**12.1**	**11.2**	**14.0**	***0.047***
Mother only	*116*	**165.2±6.9**	18.9	7.7	6.8	11.2	11.6	*0.542*
No parent	*666*	**166.4±6.4**	63.1	70.0	67.5	62.6	59.4	*0.330*
*ANOVA*		***0.017***	100% *(206)*	100% *(207)*	100% *(206)*	100% *(206)*	100% *(207)*	
DAIRY CONSUMPTION	*1028*	166.5±6.4						
Daily	*492*	**166.7±6.5**	48.1	43.9	45.9	50.0	51.5	*0.219*
Sometimes	*508*	**166.1±6.3**	51.0	54.1	52.2	46.6	43.2	*0.087*
Never	*28*	**169.6±5.5**	**1.0**	**2.0**	**2.0**	**3.4**	**5.3**	***0.014***
*ANOVA*		***0.011***	100% *(206)*	100% *(205)*	100% *(205)*	100% *(206)*	100% *(206)*	
SCHOOL LUNCHES^primary+high school^	*418*	167.0±6.2						
Daily	*272*	**168.0±6.1**	**49.3**	**59.1**	**65.9**	**69.4**	**79.1**	***0.001***
Sometimes	*74*	**165.9±5.4**	**23.2**	**19.4**	**17.6**	**17.6**	**11.6**	***0.016***
Never	*72*	**164.3±6.2**	**27.5**	**21.5**	**16.5**	**12.9**	**9.3**	***<0.001***
*ANOVA*		***<0.001***	100% *(69)*	100% *(93)*	100% *(85)*	100% *(85)*	100% *(86)*	
SCHOOL LUNCHES^high school only^	*1030*	166.5±6.5						
Daily	*363*	**167.7±6.2**	**25.1**	**34.0**	**36.0**	**39.6**	**41.5**	***0.013***
Sometimes	*295*	**166.4±6.5**	28.5	26.7	29.6	29.5	29.0	*0.383*
Never	*372*	**165.4±6.5**	**46.4**	**39.3**	**34.5**	**30.9**	**29.5**	***0.006***
*ANOVA*		***<0.001***	100% *(207)*	100% *(206)*	100% *(203)*	100% *(207)*	100% *(207)*	
PHYSICAL ACTIVITY	*1022*	166.5±6.5						
Extreme	*34*	**169.4±6.4**	1.0	2.5	5.0	2.9	5.4	*0.101*
Moderate	*599*	**166.5±6.4**	56.8	61.8	53.5	63.1	57.8	*0.823*
Low	*389*	**166.1±6.5**	42.2	35.8	41.6	34.0	36.8	*0.335*
*ANOVA*		***0.016***	100% *(206)*	100% *(204)*	100% *(202)*	100% *(206)*	100% *(204)*	

Dairy products have a direct effect on height as a source of the highest quality protein, and their significant role was particularly evident in boys (*p* < 0.001), in whom we can observe a positive correlation between daily consumption of dairy products and height in quintiles, and a negative correlation with occasional (sometimes) consumption. This finding is also very important because it confirmed the ability of the surveyed students to estimate the frequency of their dairy intake with reasonably high accuracy. The trends in females did not reach significance, but this was only due to the eccentric result in the 1 st quintile. In the remaining four quintiles, the proportion of females with daily consumption significantly increased (*p* = 0.016) and the proportion of females with occasional consumption significantly decreased (*p* = 0.015). The subcategory “never” turned out to be the least informative, as it included a negligible number of students. Furthermore, a somewhat odd finding was that females in this subcategory were unexpectedly tall. This sample was small (*n* = 28), but much larger than in males (*n* = 8), and the avoidance of dairy products had a positive relationship to body height in all quintiles (*p* = 0.014). Upon closer examination, we find that the two highest quintiles of height (*n* = 18) included six females who had been practicing veganism or vegetarianism for several years, and 12 female students who attended elite A-schools. This group of young females was, therefore, mostly highly educated and experimented with unconventional dietary regimens. For this reason, we can reasonably assume that they started experimenting with these alternative diets during their teenage years to lose weight. Thus, their current avoidance of dairy products is unlikely to represent their lifetime dietary habits.

The case of school lunches was one of the most interesting in the present study because our working hypothesis was that the system of school catering contributes to the unexpected tallness of the Czech population. In accordance with this assumption, the avoidance of school lunches since primary school had a strongly negative relationship with height in both sexes: The difference between the subcategories “daily” and “never” reached 1.9 cm in males and 3.7 cm in females. However, in the “high school only” sample, which covered only 3–4 out of 12–13 years of school attendance, school lunches ceased to be a significant factor in males, and the differences in females were attenuated.

Physical activity contributes to the healthy development of muscle mass and bones, but it is difficult to trace its effect on physical growth. In this context, the most often discussed issue is the negative impact of excessive physical activity and restricted energy intake on growth, especially in gymnasts [20]. On the other hand, it can be assumed that taller children generally have greater success in competitive sports, while obese children do not participate in sports activities, and their faster onset of puberty may lead to lower body height in adulthood [21–23]. The trend towards taller body height with more intense physical activity is indeed visible in our data and reaches statistical significance in females. Nevertheless, we do not see any close association between the degree of physical activity and height quintiles, which suggests that physical activity does not mediate the inverse relationship between height and % body fat.

The differences in height were even greater when two or three factors were combined (Supplementary Tables S4 and S5), although they did not always reach significance because the number of individuals in some combinations was too small. Linear mixed-effects models (Table 9) offer a more objective view of the statistical significance of the observed relationships because the effect of all variables is adjusted for each other. The findings do not indicate a significant effect of place of residence on height, although a slight negative trend was noted among males. Parental education had no significant role in males, but interestingly, maternal college education appared to negatively impact female height, contradicting trends observed in both sexes during the survey's first phase. A possible explanation for this anomaly may be sample size limitations, as the female sample in question contained the lowest number of individuals of all comparisons.

**Table 9:** Linear mixed-effect models of body height

Variable	Subcategories	(1) School lunches: primary and high school				(2) School lunches: high school only			
		Males (intercept: 178.26 cm)		Females (intercept: 163.89 cm)		Males (intercept: 180.10 cm)		Females (intercept: 165.43 cm)	
		Estimate	*p-*value	Estimate	*p-*value	Estimate	*p-*value	Estimate	*p-*value
Place of residence	> 100,000 inhabitants	*0.00		*0.00		*0.00		*0.00	
	5000–100,000 inhab	− 1.04	*0.110*	0.16	*0.824*	− 1.08	*0.098*	− 0.13	*0.862*
	0–5000 inhabitants	− 0.91	*0.151*	− 0.34	*0.643*	− 1.01	*0.112*	− 0.54	*0.468*
Parental education (college)	Both parents	0.20	*0.762*	0.53	*0.396*	0.34	*0.613*	0.54	*0.393*
	Father only	0.83	*0.228*	0.23	*0.725*	0.91	*0.190*	0.32	*0.623*
	Mother only	0.79	*0.245*	**− 1.82**	***0.005***	0.86	*0.204*	**− 1.69**	***0.010***
	No parent	*0.00		*0.00		*0.00		*0.00	
Dairy consumption	Daily	**1.85**	**< *0.001***	0.59	*0.146*	**1.84**	**< *0.001***	0.54	*0.183*
	Sometimes	*0.00		*0.00		*0.00		*0.00	
School lunches	Daily	1.73	*0.055*	**4.02**	**< *0.001***	− 0.19	*0.713*	**2.33**	**< *0.001***
	Sometimes	1.01	*0.382*	1.79	*0.092*	− 0.67	*0.259*	1.04	*0.038*
	Never	*0.00		*0.00		*0.00		*0.00	

The models tested two variants of responses to school lunch attendance: relevant to both primary school and high school, and relevant to high school only. Note that the subcategory ‘Dairy consumption: Never’ was excluded because the number of students was too small. Significant intergroup differences are highlighted in bold

^*^This parameter (intercept) represents a standard within each variable and is set to zero. The sum of values from individual subcategories can predict theoretical values of body height, relative to the intercept. For example, a male from a large city with > 100,000 inhabitants (0.00 cm), with both college-educated parents (+ 0.20 cm), with daily dairy consumption (+ 1.85 cm), and daily attendance of school lunches since primary school (+ 1.73 cm) would be 182.04 cm tall (178.26 + 3.78 cm = 182.04)

Nutritional factors displayed more definitive results: a positive association between daily dairy consumption and height in males, and between regular school lunch attendance and height in females. The most significant benefits of school lunches were noted in individuals who attended them daily in both primary and secondary school, with an average height increase of 1.7 cm expected for men and 4.0 cm for women. In accordance with Table 8, daily visits to school canteens in high school had a smaller effect: Although it is still significant in females (2.3 cm), the observed trends in males tend to be even negative. The absence of any positive effect in males would make sense in the context of the significant role of dairy consumption, as it suggests that the diet provided in school canteens may not be comparably effective for rapid pubertal growth.

#### Body fat vs. lifestyle

Information on % body fat was available for 2030 individuals (1002 males and 1028 females) aged 18–22 years, who filled out a questionnaire during the second phase of the survey. The relationships between % body fat and five lifestyle factors are displayed in Table 10 and Supplementary Fig. 7 and 8, and reveal multiple significant associations. In contrast to Fig. 3A, B, the inverse relationship between height and the quintiles of % body fat was significant in females and approached significance in males. As expected, the biggest differences can be seen in the variable “physical activity”: males with extreme levels of physical activity had 8.1% less body fat than males with low levels of physical activity and in women, this difference reached 9.3%. Interestingly, the differences were quite large across all three individual subcategories and were also reflected in significant trends across the quintiles of % body fat. Obviously, males were much more physically active than females, because 23.7% of males and 38.2% of females reported a low level of physical activity.

**Table 10:** Average percentage of individuals with selected lifestyle characteristics in each quintile of % body fat

**MALES**	***n***	**% body fat**	**Quintiles of % body fat in males **					***p* for trend**
			**3.00-9.62**	**9.64-12.62**	**12.63-16.16**	**16.16-22.00**	**22.07-52.52**	
Body height (cm)	*1002*		182.8±6.8	180.0±6.5	179.6±6.4	178.9±6.5	179.1±7.5	*0.066*
PLACE OF RESIDENCE	*1000*	15.95±7.61						
>100 000 inhabitants	*155*	**14.53±6.40**	17.5	14.5	19.5	14.4	11.6	*0.269*
5000-100 000 inhab.	*374*	**15.95±7.77**	37.5	39.5	33.0	41.8	35.2	*0.863*
0-5000 inhabitants	*471*	**16.41±7.81**	45.0	46.0	47.5	43.8	53.3	*0.273*
*ANOVA*		***0.028***	100% *(200)*	100% *(200)*	100% *(200)*	100% *(201)*	100% *(199)*	
PARENTAL EDUCATION (college)	*998*	15.95±7.59						
Both parents	*129*	**14.07±6.91**	**18.7**	**14.4**	**13.6**	**8.5**	**9.5**	***0.019***
Father only	*118*	**15.10±7.67**	13.6	15.4	9.5	10.9	9.5	*0.132*
Mother only	*121*	**15.55±6.97**	13.1	10.9	11.1	13.9	11.6	*1.000*
No parent	*630*	**16.57±7.75**	**54.5**	**59.2**	**65.8**	**66.7**	**69.3**	***0.008***
*ANOVA*		***0.003***	100% *(198)*	100% *(201)*	100% *(199)*	100% *(201)*	100% *(199)*	
DAIRY CONSUMPTION	*997*	15.95±7.58						
Daily	*464*	**15.13±7.34**	**57.9**	**46.3**	**45.0**	**43.8**	**39.9**	***0.037***
Sometimes	*525*	**16.62±7.74**	**42.1**	**53.2**	**54.0**	**55.2**	**58.6**	***0.044***
Never	*8*	**19.42±6.55**	**0.0**	**0.5**	**1.0**	**1.0**	**1.5**	***0.006***
*ANOVA*		***0.004***	100% *(197)*	100% *(201)*	100% *(200)*	100% *(201)*	100% *(198)*	
SCHOOL LUNCHES^primary+high school^	*546*	15.58±7.36						
Daily	*406*	**15.06±7.09**	**81.5**	**76.9**	**75.4**	**69.2**	**67.3**	***0.002***
Sometimes	*72*	**16.51±7.64**	12.6	8.7	12.7	14.4	17.8	*0.126*
Never	*68*	**17.68±8.37**	5.9	14.4	11.9	16.3	14.9	*0.131*
*ANOVA*		***0.013***	100% *(119)*	100% *(104)*	100% *(118)*	100% *(104)*	100% *(101)*	
SCHOOL LUNCHES^high school only^	*999*	15.94±7.59						
Daily	*467*	**14.94±7.01**	**55.3**	**48.3**	**51.0**	**41.5**	**37.7**	***0.021***
Sometimes	*236*	**16.41±7.94**	23.6	21.4	21.0	23.5	28.6	*0.253*
Never	*296*	**17.14±7.98**	21.1	30.3	28.0	35.0	33.7	*0.064*
*ANOVA*		***<0.001***	100% *(199)*	100% *(201)*	100% *(200)*	100% *(200)*	100% *(199)*	
PHYSICAL ACTIVITY	*982*	15.91±7.56						
Extreme	*114*	**11.95±4.27**	**16.8**	**19.8**	**13.3**	**7.1**	**1.0**	***0.025***
Moderate	*635*	**15.11±6.79**	71.1	67.0	65.8	68.7	50.5	*0.130*
Low	*233*	**20.04±8.94**	**12.2**	**13.2**	**20.9**	**24.2**	**48.5**	***0.038***
*ANOVA*		***<0.001***	100% *(197)*	100% *(197)*	100% *(196)*	100% *(198)*	100% *(194)*	

**FEMALES**	***n***	**% body fat**	**Quintiles of % body fat in females**					***p for trend***
			**5.79-20.60**	**20.61-24.17**	**24.22-28.06**	**28.12-33.03**	**33.10-57.37**	
Body height (cm)	*1028*		**167.7±6.6**	**167.0±6.3**	**166.0±6.1**	**166.6±6.5**	**165.1±6.5**	***0.045***
PLACE OF RESIDENCE	*1025*	27.06±7.52						
>100 000 inhabitants	*92*	25.63±6.93	**10.7**	**10.2**	**9.2**	**7.9**	**6.8**	***0.002***
5000-100 000 inhab.	*445*	26.80±7.52	48.1	40.0	43.7	41.9	43.4	*0.513*
0-5000 inhabitants	*488*	27.56±7.60	41.3	49.8	47.1	50.2	49.8	*0.158*
*ANOVA*		*0.050*	100% *(206)*	100% *(205)*	100% *(206)*	100% *(203)*	100% *(205)*	
PARENTAL EDUCATION (college)	*1026*	27.08±7.53						
Both parents	*128*	**24.70±6.57**	**19.9**	**15.7**	**9.3**	**9.8**	**7.8**	***0.022***
Father only	*118*	**26.43±7.64**	13.6	10.3	11.7	10.7	11.2	*0.343*
Mother only	*115*	**25.52±6.64**	11.7	14.7	14.6	6.8	8.3	*0.242*
No parent	*665*	**27.92±7.70**	**54.9**	**59.3**	**64.4**	**72.7**	**72.8**	***0.005***
*ANOVA*		***<0.001***	100% *(206)*	100% *(204)*	100% *(205)*	100% *(205)*	100% *(206)*	
DAIRY CONSUMPTION	*1022*	27.08±7.54						
Daily	*487*	26.68±7.47	**51.2**	**52.2**	**45.5**	**45.9**	**43.4**	***0.035***
Sometimes	*507*	27.58±7.65	**45.9**	**44.4**	**50.0**	**53.7**	**54.1**	***0.025***
Never	*28*	25.19±5.95	2.9	3.4	4.5	0.5	2.4	*0.480*
*ANOVA*		*0.067*	100% *(205)*	100% *(205)*	100% *(202)*	100% *(205)*	100% *(205)*	
SCHOOL LUNCHES^primary+high school^	*413*	26.61±7.07						
Daily	*269*	**25.77±6.57**	**76.8**	**65.5**	**65.2**	**59.8**	**57.4**	***0.017***
Sometimes	*73*	**27.03±6.77**	13.4	21.8	14.6	20.7	17.6	*0.606*
Never	*71*	**29.34±8.45**	**9.8**	**12.6**	**20.2**	**19.5**	**25.0**	***0.009***
*ANOVA*		***<0.001***	100% *(82)*	100% *(87)*	100% *(89)*	100% *(87)*	100% *(68)*	
SCHOOL LUNCHES^high school only^	*1024*	27.09±7.54						
Daily	*360*	**26.03±7.05**	42.4	34.3	36.8	34.6	27.7	*0.055*
Sometimes	*294*	**26.60±7.03**	27.8	34.3	29.9	25.9	25.7	*0.326*
Never	*370*	**28.51±8.16**	**29.8**	**31.4**	**33.3**	**39.5**	**46.6**	***0.012***
*ANOVA*		***<0.001***	100% *(205)*	100% *(204)*	100% *(204)*	100% *(205)*	100% *(206)*	
PHYSICAL ACTIVITY	*1016*							
Extreme	*32*	**19.92±5.54**	**9.0**	**3.4**	**2.0**	**1.5**	**0.0**	***0.033***
Moderate	*596*	**26.18±7.10**	**67.5**	**61.8**	**64.4**	**53.4**	**46.6**	***0.024***
Low	*388*	**29.19±7.69**	**23.5**	**34.8**	**33.7**	**45.1**	**53.4**	***0.008***
*ANOVA*		***<0.001***	100% *(200)*	100% *(204)*	100% *(202)*	100% *(204)*	100% *(206)*	

The sample incorporates 2030 individuals (1002 males and 1028 females) aged 18–22 years who underwent measurements of body composition and filled out a questionnaire. Significant intergroup differences are highlighted in bold

Other variables showed a smaller importance, although the differences were mostly highly significant, especially in females. Of particular note is the detrimental role of low parental education, which strongly influenced the trend in % body fat quintiles in both sexes. Daily consumption of dairy products had a positive association with a lean body build, but occasional consumption was already correlated negatively in both sexes. The low % body fat in females who avoid consuming dairy products again supports the idea that they were experimenting with weight loss diets. Daily attendance of school lunches was significantly correlated with lower % body fat in males, whereas the situation observed in females leaves the impression that they benefit from any attendance, even occasional.

Intergroup differences in % body fat progressively increased when more factors were combined (Supplementary Tables S6–S8), which replicates the findings from the analysis of body height, but sample sizes were again suboptimal in some combinations. The results of linear mixed-effect models (Table 11) were quite consistent in both sexes, with the strongest effect of physical activity. Parental education and school lunch attendance were the second most important factors, especially in females, and emphasize the importance of family background and a regular eating regimen. Daily school lunch attendance had the strongest impact on % body fat when it had been maintained since primary school. Daily consumption of dairy products had a smaller role, although it was always positive, and approached significance in males.

**Table 11:** Linear mixed-effect models of % body fat

Variable	Subcategories	(1) School lunches: primary & high school				(2) School lunches: high school only			
		Males (Intercept: 21.48%)		Females (Intercept: 31.56%)		Males (Intercept: 20.43%)		Females (Intercept: 30.62%)	
		Estimate	*p-*value	Estimate	*p-*value	Estimate	*p-*value	Estimate	*p-*value
Place of residence	> 100,000 inhabitants	*0.00		*0.00		*0.00		*0.00	
	5000–100,000 inhab	0.98	0.153	0.21	0.798	1.08	0.118	0.39	0.642
	0–5000 inhabitants	1.09	0.110	0.40	0.630	1.16	0.088	0.46	0.579
Parental education (college)	Both parents	**− 1.94**	**0.006**	**− 2.32**	**0.001**	**− 1.87**	**0.008**	**− 2.25**	**0.002**
	Father only	− 0.95	0.194	− 1.00	0.169	− 0.87	0.235	− 1.01	0.166
	Mother only	− 0.21	0.774	− 1.70	0.022	− 0.15	0.830	− 1.72	0.020
	No parent	*0.00		*0.00		*0.00		*0.00	
Dairy consumption	Daily	− 0.88	0.058	− 0.60	0.189	− 0.86	0.066	− 0.55	0.229
	Sometimes	*0.00		*0.00		*0.00		*0.00	
School lunches	Daily	**− 2.19**	**0.022**	**− 2.76**	**0.005**	**− 1.39**	**0.012**	**− 1.85**	**0.001**
	Sometimes	− 1.54	0.206	− 1.86	0.124	− 0.31	0.620	− 1.39	0.014
	Never	*0.00		*0.00		*0.00		*0.00	
Physical activity	Extreme	**− 8.49**	**< 0.001**	**− 8.56**	**< 0.001**	**− 8.38**	**< 0.001**	**− 8.51**	**< 0.001**
	Moderate	**− 4.86**	**< 0.001**	**− 2.85**	**< 0.001**	**− 4.81**	**< 0.001**	**− 2.86**	**< 0.001**
	Low	*0.00		*0.00		*0.00		*0.00	

The models tested two variants of responses to school lunch attendance: relevant to both primary school and high school, and relevant to high school only. Note that the subcategory ‘Dairy consumption: Never’ was excluded because the number of students was too small. Significant intergroup differences are highlighted in bold

^*^This parameter (intercept) represents a standard within each variable and is set to zero. The sum of values from individual subcategories can predict theoretical values of % body fat, relative to the intercept. For example, a male from a large city with > 100,000 inhabitants (0.00%), with both college-educated parents (− 1.94%), with daily dairy consumption (− 0.88%), daily attendance of school lunches (− 2.19%), and extreme physical activity (− 8.49%) would have 7.98% body fat (21.48–13.5 = 7.98)

The place of residence showed no significant association with body fat, but it is noteworthy that in all four comparisons, there was a consistent trend towards slightly higher % body fat in students coming from small settlements. The same situation can be seen in Table 10, where ANOVA identified weakly significant differences among the subgroups of males and borderline significant differences in females. This finding would seem counterintuitive at first glance, but it may reflect the current reality of activity patterns, when children from big cities have more opportunities for physical activity than children from villages, who lag behind urban children in terms of physical fitness [24].

#### Social background vs. lifestyle

Table 12 illustrates the relationship between parental education and the three other examined variables (dairy consumption, school lunches, physical activity). Apparently, the college education of parents influences the everyday diet of students (dairy consumption and school lunches), as in three of the four “daily” subcategories, we observe increasing trends regarding parental education (None > One > Both). At the same time, school lunch attendance decreases with age: in primary school, 8.5% of males and 10.8% of females do not attend school lunches, but in high school, these numbers increase to 29.7% in males and 36.1% in females. Simultaneously, the percentage of daily visitors decreases from 71.9% to 46.7% in males and from 69.4 to 35.2% in females. Interestingly, contrary to our expectations, parental education does not have a clear relationship to physical activity.

**Table 12:** Proportion (%) of males and females in the subcategories of dairy consumption, school lunches (primary + high school), and physical activity, according to parental education

**Dairy consumption**	**Parental education (college): Males *(n =1002)***					**Parental education (college): Females ***(n =1026)*				
	**Both**	**Father**	**Mother**	**One**	**None**	**Both**	**Father**	**Mother**	**One**	**None**
Daily	*63.6*	*54.2*	*42.6*	*48.3*	*42.5*	*50.8*	*50.4*	*53.4*	*51.9*	*45.8*
Sometimes	*36.4*	*44.9*	*57.4*	*51.3*	*56.3*	*44.7*	*45.3*	*45.7*	*45.5*	*51.7*
Never	*0.0*	*0.8*	*0.0*	*0.4*	*1.1*	*4.5*	*4.3*	*0.9*	*2.6*	*2.4*

**School lunches**	**Parental education (college): Males** ***(n =546)***					**Parental education (college): Females** ***(n =418)***				
	**Both**	**Father**	**Mother**	**One**	**None**	**Both**	**Father**	**Mother**	**One**	**None**
Daily	*87.3*	*87.5*	*75.4*	*81.9*	*67.6*	*85.7*	*77.6*	*82.0*	*79.6*	*53.4*
Sometimes	*8.9*	*5.0*	*13.0*	*8.7*	*16.4*	*7.9*	*13.8*	*18.0*	*15.7*	*21.1*
Never	*3.8*	*7.5*	*11.6*	*9.4*	*16.0*	*6.3*	*8.6*	*0.0*	*4.6*	*25.5*

**Physical activity**	**Parental education (college): Males* (n =988)***					**Parental education (college): Females** ***(n =1020)***				
	**Both**	**Father**	**Mother**	**One**	**None**	**Both**	**Father**	**Mother**	**One**	**None**
Extreme	*15.9*	*12.9*	*20.7*	*16.9*	*9.7*	*7.8*	*2.5*	*3.4*	*3.0*	*2.6*
Moderate	*59.8*	*64.7*	*60.3*	*63.4*	*65.8*	*58.9*	*61.9*	*66.4*	*64.1*	*56.6*
Low	*24.2*	*22.4*	*19.0*	*20.7*	*24.6*	*33.3*	*35.6*	*30.2*	*32.9*	*40.8*

#### Vegans/vegetarians

The proportion of vegans or vegetarians in the sample examined during the second phase was very low. Only one male out of 1011 (0.1%) and 14 females out of 1034 (1.4%) reported practicing a meat-free diet (0.7% of the total sample), and three females were vegan or “nearly vegan”. However, only three females were experimenting with a meat-free diet for more than 2 years. It is, therefore, understandable that no conclusions can be drawn from the height and body composition of these individuals. The sample of 14 females was tall (169.8 ± 4.4 cm), but overweight (29.13 ± 8.13% body fat), suggesting that the primary motive for this type of dieting was to lose excess body fat. The three females who reported a longer practice of vegetarianism (6 and 9 years, and lifelong) were also tall (169.0, 168.0, and 174.2 cm), but were among the most overweight in this sample (37.31%, 36.79%, and 31.17% body fat).

#### Chronic disease or serious injury

Out of 1002 males, who responded to the question concerning chronic diseases during their life, 263 were affected and their average height (179.1 ± 6.8 cm) was significantly lower (ANOVA *p* = 0.006) than in 739 males, who did not report any chronic diseases (180.5 ± 6.9 cm). However, there was no difference (ANOVA *p* = 0.894) between 181 males, who reported a serious injury during their life (180.1 ± 6.7 cm) and 825 males, who reported no serious injury (180.1 ± 6.9 cm). Likewise, no significant difference (ANOVA *p* = 0.504) was found between 313 females with the experience of chronic diseases (166.7 ± 6.6 cm) and 719 females without chronic diseases (166.4 ± 6.4 cm). Serious injuries in females played no role either, as there was no difference (ANOVA *p* = 0.778) between 136 females with past injuries (166.3 ± 6.1 cm) and 894 females without serious injuries (166.5 ± 6.5 cm).

## Discussion

### The current state of the height trend

The possibilities of this study to assess the current state of the body height trend in the Czech Republic were limited from the beginning, given that the research took place in only 4 out of 14 regions, and due to the COVID epidemic, it has remained unfinished. Nevertheless, it is still the most extensive anthropological research of young adults that has taken place in the country since 2001. The most representative data from the Brno-City District show that, compared to the height of 18-year-olds in CAV 2001 [1], young males are 0.3 cm taller (180.5 cm), but young females are 0.8 cm shorter (166.5 cm). This was an unexpected finding, and the surprisingly low female average was also one of the reasons why we proceeded to expand the sample size, as we believed that it was not sufficient. Despite that, this result did not change and was subsequently confirmed in all the surrounding regions. As a result, the male–female difference in the present study (14.0 cm) is substantially greater than in CAV 2001 (12.9 cm).

These discrepancies are quite fundamental and are not easy to explain, especially since other available research does not show a decrease in body height in young women. Perhaps the most likely reason may lie in the fact that previous CAV surveys preferred schools with 4-year courses in order to obtain large sample sizes (J. Vignerová—personal communication). Since the vocational C1 and C2 schools include a large proportion of 3-year courses, it is inevitable that they were underrepresented (or almost completely omitted) and the average height was probably overestimated. Assuming that the male–female difference in 2001 was ~ 13 cm, we can speculate that the true height of young adults was only 179.5 cm in men and 166.5 cm in women. Interestingly, the first figure agrees with the contemporary study by Jirkovský [5], who measured conscripts coming predominantly from three Bohemian regions. Provided that the height of males has further increased and the height of females has stagnated, we could also explain why the current male–female difference is ~ 1 cm higher.

The data from the second phase suggest that male height varies more geographically than female height, which would be understandable because males naturally have higher requirements for nutrients than females during physical growth [25] and hence respond more sensitively to environmental conditions. Although the samples from the Oloumouc, Vysočina, and Zlín regions were too small to draw any definitive conclusions, it is interesting that men from the area of Zlín (then Gottwaldov) were the shortest in the Czech part of Czechoslovakia already in the past, during the measurement of participants in the mass public sports events (Spartakiads). Men from Prague and the area of Plzeň were the tallest [26] (Supplementary Fig. S9). This situation would have an economic explanation, as the median wage in the current Zlín region was the second lowest in the country in 2019, after the Karlovy Vary region, which is chronically characterized by the least favorable socio-economic statistics. The South Moravian region held 5th place in this comparison, the Vysočina region was in 9th place, and the Olomouc region in 12th place out of 14 regions. The highest median wages can be found in the capital of Prague, the Central Bohemian region, and the Plzeň region [27]. Assuming that the economic situation in regions influences body height, the average height obtained in the present study from four predominantly Moravian regions would probably slightly underestimate the overall average for the Czech Republic. The average height found in the South Moravian region, which is characterized by moderate economic statistics, may therefore be very close to the national average.

### Height trend in the context of nutrition

The height trend in the Czech Republic can be further placed in the context of changing dietary protein quality. According to the Czech Statistical Committee [28], the economic transformation in the early 1990 s had a very significant impact on the eating habits of Czechs: The consumption (supply) of high-quality protein sources (dairy products, pork, beef, eggs) decreased due to relatively high costs and was replaced by cereals and poultry (Supplementary Fig. S10A). Similar trends in protein supply are documented by the international FAOSTAT statistics [29] (Supplementary Fig. S10B). This dietary change inevitably led to a profound decline in the average protein quality, which can be expressed by the “protein index”—the ratio between the most consumed proteins of the highest and lowest quality in the FAOSTAT database (dairy and pork/wheat). This index is the strongest ecological correlate of male height in 44 European countries (*r* = 0.62, *p* < 0.001) [2]. Its values dramatically decreased after 1993 and started to increase again only after 2014. In 2019, they surpassed the level from 1993 (Supplementary Fig. S10C). Nevertheless, the trends according to the Czech Statistical Committee and FAOSTAT are not completely identical, and the decline in the intake of wheat in the FAOSTAT database is relatively more rapid. The causes of this discrepancy are unclear.

Interestingly, very similar changes in the protein index occurred in Denmark, where the height of conscripts decreased from 180.4 cm to 179.8 cm between 1990 and 2011 [30]. After this period, protein quality started to increase again due to a rapidly rising intake of dairy products, and Danish conscripts reached 181.7 cm in 2020. Given that the initial height of Czech and Danish males was very similar, one could expect the same negative development in the Czech Republic as well, especially considering the fact that our samples were measured mainly in the period 2015–2017, i.e., before the improvement in protein quality could manifest significantly. However, none of the available data indicate a downward trend in the youngest generation. Actually, the supposed decline in stature should already be evident in CAV 2001, yet both male and female averages in this survey are higher than in CAV 1991 [1]. Even more perplexing is the fact that young Czechs are taller than their peers in Germany and Austria (cf. [2]), countries with a much higher GDP per capita and a significantly higher average protein quality.

All these findings again lead to interesting questions regarding the beneficial role of the school catering system, because young adults, who have visited school canteens daily since primary school, are taller than those who do not visit school canteens at all. Nevertheless, this hypothesis does not find unequivocal support in our data, as the benefits of school lunch attendance are much more evident in females, whose height is in all likelihood stagnating. Still, the average height gain of 1.7 cm observed in males (albeit only in a subgroup with consistent eating habits since primary school) would more than offset the negative impact of declining dietary protein quality seen in Denmark. In any case, to assess this effect more objectively, it would be desirable to have similar data available from neighboring countries.

### Lifestyle strongly influences body height and body composition, despite high social equality

Our survey has documented very significant trends in height and body composition across various subcategories of lifestyle factors. Since these lifestyle factors have a meaningful causal impact, the observed relationships can also be considered causal, although it is understandable that it is not possible to completely control for the complex “matrix” of behaviors that may influence the significance of these relationships. For example, the daily attendance of school lunches, which is typical of children from highly educated families, may be associated with higher quality nutrition in the family, while children from uneducated families, who ignore school lunches, may generally consume poor-quality foods. For this reason, it was necessary to use linear mixed-effect models, which could more or less eliminate the interaction of these environmental factors. However, the causal role of school lunches is already strengthened by the simple fact that their observed effect on height and % body fat is roughly twice as strong when their attendance at high school is combined with attendance at basic school.

In any case, our data clearly demonstrate that students from highly educated, well-nourished social groups with healthy lifestyle habits are taller and leaner. In fact, the linear mixed-effect models show that in the combined primary school and high school sample, daily visitors to school lunches who consume dairy products daily and come from college-educated families can theoretically exceed their classmates from the opposite spectrum of lifestyle factors by 3.8 cm in males and 5.1 cm in females. This is remarkable given that the average value of the Gini index (a measure of social inequality) in the Czech Republic was the 2nd lowest in the world between 2000 and 2019 (after Slovenia and on a par with Slovakia) [31]. Differences in % body fat between the same social extremes would reach 5.0% in males and 5.7% in females, but they would be greatly overshadowed by the effect of extreme physical activity, which was associated with 8.5% and 8.6% less body fat in males and females, respectively. The difference between students with low and moderate physical activity (4.9% for males, 4.8% for females) would indicate that even moderate physical activity has a very positive effect on body fat reduction. However, the literature also points out the possibility of reverse causality: obese children avoid physical activity precisely because they are obese [32]. How this factor was reflected in our data is not possible to say, since our questionnaires did not contain questions regarding personal relationship to sport. This issue could be addressed in our future research.

The relationship between socio-economic factors and obesity in the Czech Republic agrees with the trends observed in other developed countries, where the highest prevalence of obesity can be found in lower social groups. In contrast, the situation in developing countries is quite the opposite, and higher social classes have the highest obesity rates [33, 34]. Understanding these paradoxical relationships is not difficult if we take into account that lower social classes in developing countries suffer from malnutrition, whereas higher social classes in developing countries and lower social classes in developed countries have an abundance of low-quality, cheap food with a high proportion of carbohydrates and cereals as the major protein source. This naturally leads to a high dietary glycemic load and creates the conditions for easy weight gain. On the other hand, higher social classes in developed countries consume more quality animal-based foods and have generally healthier eating habits, which were also demonstrated in the present study.

A more concrete list of factors that explain the positive relationship between low social status and obesity in affluent countries was recently summarized in a review by Gebremariam et al. [35]: Higher consumption of sugar-sweetened beverages, more time spent in front of the TV and computer, higher BMI of parents, shorter duration of breastfeeding, irregular breakfast consumption, maternal smoking during pregnancy, and unhealthy infant feeding practices.

### Inverse relationship between height and % body fat

The inverse relationship between social status and obesity in developed countries is analogous to the inverse relationship between height and % body fat (obesity, BMI) documented in the present study. This phenomenon obviously represents a common biological pattern because it has been reported even by many other studies [36–38] and is actually curvilinear in the global context: inverse in well-nourished populations and positive in poorly nourished populations [39] (Supplementary Fig. S11A–S11B). It is therefore inevitable that as nutrition improves, the positive relationship changes to a negative one, as documented in Switzerland over the last 150 years [38]. These data cast doubt on attempts at genetic or anthropological (allometric) explanations and suggest that the causes should be sought primarily in nutritional factors, which are directly related to physical growth. If there is any anthropological factor contributing to the inverse height-body fat relationship, it is the disproportionately increasing percentage of muscle mass in taller individuals, a consequence of more elongated body proportions (Supplementary Table 9).

The role of dietary habits during the growth period can be supported by an observational study conducted in Brazil, where the link between short stature and high BMI was the strongest in young adults and progressively decreased with increasing age [40]. Similarly, longitudinal data from the USA show that the recent obesity epidemic has disproportionately affected tall individuals and the inverse height/BMI relationship was markedly attenuated in women and disappeared in men [41]. This development also undermines the hypothesis that obesity in short individuals is caused by excess calories obtained from average serving sizes in restaurants.

Judging from our data, the relationship between height and % body fat is unaffected by physical activity, as there were no differences in physical activity patterns within each height quintile. In fact, the partial correlation of height with % body fat in both males (r = -0.13) and females (r = -0.13, *p* < 0.001) remains practically the same even after adjusting for physical activity level. The only lifestyle factor that reduces this partial correlation (to r = 0.07, *p* = 0.18) is females' attendance at school lunches from basic school onwards. The cumulative effect of both dietary factors (dairy consumption and school lunch attendance) during basic and high school would be + 3.6 cm and −3.1% in males, and + 4.6 cm and − 3.4% in females. In other words, a diet based on high-quality animal proteins will not only lead to rapid growth, but will also guarantee a low glycemic load, which is an important prerequisite for obesity prevention. Such a diet also ensures the maximum utilization of the nutrients consumed because if some essential amino acid is not received in food in sufficient quantity, protein synthesis is impaired, excess amino acids are oxidized [42], and used for energy or stored as subcutaneous fat. In contrast, a plant-based diet is less nutritionally dense, deficient in multiple essential amino acids (especially lysine and methionine & cysteine), and requires a higher protein and energy intake. It is therefore clear that this style of eating cannot be considered wise in children, as it will lead to either malnutrition or obesity.

These conclusions can be well aligned with the current state of knowledge. Although it seems that high-protein diets may promote the development of obesity in preschool children, this changes in school-age children and adolescents, in whom a beneficial effect of such diets on obesity and lean body mass is usually observed [43, 44]. Our ecological data based on 136 world populations [2] particularly highlight the growth-stimulating effect of dairy products, pork, and eggs, which can be supplemented by beef and marine fish. In contrast, poultry has a negative relationship with height in developed countries, and cereals and legumes have a universally negative association with physical growth worldwide. In addition, refined cereals are also the source of high-glycemic carbohydrates. It cannot be ignored that the extremely high prevalence of child obesity in the USA, the Caribbean, and in the Muslim countries of North Africa and the Near East (see again Supplementary Fig. S11A–S11B) is accompanied by a very similar dietary pattern characterized by the reliance on cereals and poultry as the main sources of protein. On the other hand, the consumption of red meat (beef, pork) has been decreasing or is very low due to religious reasons (the prohibition on pork in Muslim countries) (Supplementary Figs. S12A–S12C).

A notable age-related exception to the inverse relationship between height and % body fat is children at the onset of puberty. As already mentioned [21–23], obese children mature faster and are markedly taller than lean children during the pre-pubertal age, but their preterm skeletal maturity may eventually lead to somewhat shorter stature. Such a pattern can be found even in a sample of children aged 9–12 years from the Brno-City District, who were measured by a different team from our institution between 2018 and 2021: the relationship between body height and % body fat was neutral in 272 boys (*r* = 0.07, *p* = 0.24), but positive in 315 girls (*r* = 0.19, *p* < 0.001). Unfortunately, no socio-economic or dietary information was collected during this research and hence, it cannot be compared with our data.

### Implications for child nutrition and obesity prevention

The results of this study also have important implications for the upcoming reform of the school catering system in the Czech Republic, which aims to follow in the footsteps of Western countries and promotes plant-based diets at the expense of animal products, especially red meat. The authors of this reform believe that such a dietary change will lead to an overall improvement in children’s diets and the prevention of child obesity. However, it should be noted that commonly implemented nutritional interventions in children have not yet yielded the desired results in terms of obesity prevention [45]. Usually, they contain stereotypical formulas encouraging the consumption of fruits and vegetables, without taking into account that these food components are marginal and, therefore, cannot fundamentally influence the nature of the children’s diet. The most recent review [46] concluded that there exists some evidence for the association between child obesity and sugar-sweetened beverages, skipping breakfasts, irregular family meals, a lower number of daily meals, and larger portions of high-energy foods. The authors generally recommended the “Mediterranean” dietary pattern based on expert opinion, which is, however, highly debatable as Mediterranean countries reach the highest prevalence of child obesity among OECD countries [11].

In recent years, these preventive strategies have also been influenced by environmental efforts for global sustainability and further emphasize limiting the consumption of animal products. In the context of the findings from this study, it is clear that this approach is completely misguided. In fact, several Western countries (USA, UK, Netherlands, Denmark) that have implemented this policy and reduced the consumption of red meat (at the expense of cereals and poultry) have already seen a decline in height among the young generation [30]. Since the estimated daily protein intake in European children aged 2–9 years is ~ 2.7 g/day [47], which is 2–3 times higher than the recommended intake of ~ 0.7–1.0 g complete proteins/day [44], it is clear that current recommendations for protein intake in children greatly underestimate actual needs. Further government-directed increases in the proportion of low-quality plant proteins will inevitably have dire consequences for children’s health. In addition to suboptimal physical growth, the inverse height/% body fat relationship predicts that these policies will paradoxically support the increase in child obesity and they may also lead to a decrease in population IQ scores because brain development critically depends on the intake of important nutrients [48, 49].

An illustrative example of the failure of such government programs is the British *Food for Life project*, which was recently presented in the Czech Republic by public initiatives as an example that school canteens should follow. This project was implemented in more than 7500 British schools [50] and is based on a nutritional philosophy that calls for a reduction in meat consumption [51]. Despite advertising claims of alleged benefits, the objective fact is that the proportion of overweight English children aged 10–11 years continues to rise, and developments following the COVID-19 pandemic suggest that this upward trend has not changed [52]. In addition, the positive trend in height among young English adults has reversed after more than 100 years of continuous growth [30, 53]. This negative development is clearly reflected in the FAOSTAT statistics, which show that the supply of cereals in the British diet has increased by ~ 50% since the early 1980s, while red meat supply has fallen slightly.

The roots of the “plant-based” nutrition policies can be traced back to the 1970s, when a hasty political decision in the USA, without sufficient evidence, led to the demonization of saturated fat and animal foods in general [54–58]. The legacy of these dietary guidelines is still perpetuated by heavily confounded observational studies linking red meat to virtually every disease imaginable, including obesity, without a meaningful mechanistic explanation (cf. [59–62]). At the same time, these supposed associations cannot be confirmed by controlled clinical trials [63–66], are in irreconcilable contradiction with the most elementary health and nutrition statistics [67], and do not even make sense from an evolutionary perspective [68]. Although changing the current paradigm in human nutrition will need additional evidence, such as that recently provided by the PURE study [69], the damage that the current food policy has done to children in developed countries is already so obvious that it requires an immediate response from relevant institutions.

## Conclusion

Due to the unforeseeable outbreak of the COVID epidemic, our research has not met all of its intended goals. The first research objective—mapping the current state of the height trend—has only been partially met due to the lack of sufficiently representative data from all four regions. Nevertheless, the positive fact is that our evenly stratified sample from the Brno-City District is very numerous (*n* = 2610) and enabled us to set standards for future research. The results from the three remaining regions, although incomplete, agree with the historical situation and have a normal distribution of values, which suggests that they are probably not too far from reality. Overall, these data indicate that the height trend in the Czech Republic may continue at a slow pace in males and is already stagnating in females. The second research objective—identifying environmental factors influencing height and obesity—was fulfilled more satisfactorily, and the total sample of students who took the body composition measurement (*n* = 2030) was reasonably large to draw fundamental conclusions. Their main message is the finding that body height is inversely related to % body fat, which is a pattern that is found universally in all developed countries.

Based on this evidence, we conclude that a high-quality, animal-based, low-glycemic diet should have an all-round positive effect on the physical and psychological development of children. This is particularly relevant in the context of the planned reform of the school catering system in the Czech Republic, which is clearly going in the wrong direction. It is likely that until this finding is fully accepted in international recommendations, all current strategies to reduce child obesity will not have a significant effect. On the contrary, ecologically motivated efforts to promote plant-based dietary guidelines have already had a very negative impact on children’s health in many Western countries and should be seriously reconsidered by key international institutions.

Retrospectively, it could be argued that the questionnaire used during the second phase of the survey could potentially contain more items, especially related to food consumption, but such unreliable data would not be possible to verify and we therefore wanted to avoid similar errors that accompany traditional observational studies. Dairy consumption was essentially the only food item that could realistically be reported with decent accuracy and subsequently verified by comparison with body height. Similarly, it would not be reasonable to expect that the examined students would be able to provide accurate information regarding the economic level of their families. Nevertheless, our future surveys could add questions related to fast-food consumption, the number of siblings (a factor influencing the distribution of resources within families), parental unemployment, personal attitudes to physical activity, and the specific sport that the students practice.

## Supplementary Information

Supplementary Figure S1. Average height of 17-year-old boys and girls in nationwide anthropometric surveys (CAVs) between 1951 and 2001 (statistically ‘smoothed’ values). *Source:* Vignerová et al. [4]. Supplementary Figure S2. Average height of males (*n* = 582) and females (*n* = 656) in the health survey *Physical Fitness in the Czech Republic (2011–2013).* Note that height in the oldest cohorts is also influenced by the age-related decrease in stature. *Source:* Grasgruber et al. [8]. Supplementary Figure S3. Development of the prevalence of overweight and obesity in children (age groups 5, 9, 13, and 17 years) from 1991 to 2021. The classification is based on standardized percentile graphs from the 5th CAV 1991 (90–97th percentile = overweight, ≥ 97th percentile = obese). *Sources:* 1991: 5th CAV 1991; 1996–2016: The Health of Children; 2021: Study of Anthropological Data of Czech Children (SPLDD) 2021 [9]. Supplementary Figure S4. Relationship between average male and female height in individual districts, according to the place of residence. Supplementary Figure S5. Average percentage of individuals with selected lifestyle characteristics in each quintile of body height. Supplementary Figure S6. Average percentage of individuals with selected lifestyle characteristics in each quintile of body height. Supplementary Figure S7. Average percentage of individuals with selected lifestyle characteristics in each quintile of % body fat. Supplementary Figure S8. Average percentage of individuals with selected lifestyle characteristics in each quintile of % body fat. Supplementary Figure S9. Height of participants in the Czechoslovak Spartakiads (young and middle-aged men) in 1955, 1960, and 1965 (*n* = 11387) divided regionally by major cities. The average height was 173.7 cm (*n* = 8168) for the territory of the present-day Czech Republic and 171.3 cm (*n* = 3128) for present-day Slovakia. Cited in Suchý [26]. Supplementary Figure S10. A) Food supply in the Czech Republic (1948–2023), according to the Czech Statistical Committee [28]. B) Protein supply in the Czech Republic (1992–2020) according to FAOSTAT [29]. The data precede a revision of the FAOSTAT methodology in 2021. C) Protein quality (protein index) in the Czech Republic, four neighboring countries, and Denmark (1961–2020), according to FAOSTAT [29]. Supplementary Figure S11. A) Relationship between male height and the prevalence of obesity in boys (177 populations). B) Relationship between female height and the prevalence of obesity in girls (174 populations). Data on height come from Grasgruber & Hrazdíra [2] and were supplemented by unpublished data for sub-Saharan Africa. Data on obesity rates in children aged 10–19 years were taken from the WHO website [19]. The graphs do not include Oceania, due to the existence of a highly specific fat-free mass/fat-mass relationship in Oceanian populations. Supplementary Figure S12. A) Trends in protein supply from main food items in the United States between 1961–2022. B) Trends in protein supply from main food items in Kuwait between 1961–2022. C) Trends in protein supply from main food items in the Bahamas between 1961–2022. *Source:* FAOSTAT: Food balances [29]. *Note:* FAOSTAT data for the period 1961–2013 are according to the older FAOSTAT methodology. FAOSTAT data for the period 2014–2022 are according to the new FAOSTAT methodology which was radically updated in 2021. Although the relative trends in consumption development remain the same, the absolute values may differ with the new methodology and are not always comparable with the old methodology. Supplementary Table S1. Average height in 31 individual schools measured during the first phase of the survey in the Brno-City district. Supplementary Table S2. Average height in 40 individual schools measured during the second phase of the survey in four regions. Supplementary Table S3. Average height in 16 individual districts (total sample aged 18–20 years), according to the place of residence. Supplementary Table S4. Average height in various combinations of lifestyle factors. Supplementary Table S5. Differences in average height between the extremes of various combinations of lifestyle factors. Supplementary Table S6. Average % body fat in various combinations of lifestyle factors. Supplementary Table S7. Average % body fat in various combinations of lifestyle factors. Supplementary Table S8. Differences in % body fat between the extremes of various combinations of lifestyle factors. Supplementary Table S9. Correlations between body composition and height & body proportions.

## Data Availability

The datasets used and/or analyzed during the current study are available from the corresponding author on reasonable request.
