# Correction: Body height, body composition, and lifestyle of Czech high school students: implications for the most appropriate strategies supporting physical growth and preventing obesity

**DOI:** 10.1186/s40101-026-00450-w

**Published:** 2026-10-06

**Authors:** Pavel Grasgruber, Eduard Hrazdíra, Zuzana Hlavoňová, Nikola Stračárová, Dominik Bokůvka, Jan Cacek, Jan Huleš, Tomáš Kalina, Tereza Králová, Oldřich Racek, Lenka Svobodová, Marie Svobodová

**Affiliations:** 1 Faculty of Sports Studies, Masaryk University, Kamenice 5, Brno, 625 00 Czech Republic; 2 Centre of Sports Activities, Brno University of Technology, Brno, Czech Republic

**Correction: J Physiol Anthropol 45, 8 (2026)**

**https://doi.org/10.1186/s40101-025-00418-2**

Following the publication of the original article [1], the authors identified the following errors.

1. The total number of females under C-schools (*n* = 14) in Table 4 is incorrect. It should be 359 instead of 336.
2. The markings "A" and "B" were added to Figure 3. The caption has been updated from “Fig. 3 A Relationship between body height and % body fat in males from four Czech regions, according to the level of physical activity. B Relationship between body height and % body fat in females from four Czech regions, according to the level of physical activity” to “Fig. 3 A Relationship between body height and % body fat in males from four Czech regions, by height quintile. B Relationship between body height and % body fat in females from four Czech regions, by height quintile.”
3. Table 7 was replaced.
4. Table 9 contains several typing errors (-1.06 instead of -1.08, "Daily consumption" instead of "Dairy consumption", 177.89 instead of 178.26 cm).
5. Table 11 contains a typing error ("Daily consumption" instead of "Dairy consumption").
6. Captions "FEMALES" in Table 12, which appear to be deleted during the final editing of the article, were again added to the table.
7. The original passage in the section "*Inverse relationship between height and % body fat"* has been replaced with the following sentence: "If there is any anthropological factor contributing to the inverse height-body fat relationship, it is the disproportionately increasing percentage of muscle mass in taller individuals, a consequence of more elongated body proportions (Supplementary Table 9)."

The original article [1] has been updated.
