# Supplementary material for: Body height, body composition, and lifestyle of Czech high school students: implications for the most appropriate strategies supporting physical growth and preventing obesity
Source: J Physiol Anthropol. 2026 Mar 9;45:8. doi: 10.1186/s40101-025-00418-2 (PMC13045034; doi:10.1186/s40101-025-00418-2)
Supplement: Supplementary file 1 — Supplementary Figure S1. Average height of 17-year-old boys and girls in nationwide anthropometric surveys (CAVs) between 1951 and 2001 (statistically ‘smoothed’ values). Source: Vignerová et al. [4]. Supplementary Figure S2. Average height of males (n = 582) and females (n = 656) in the health survey Physical Fitness in the Czech Republic (2011–2013). Note that height in the oldest cohorts is also influenced by the age-related decrease in stature. Source: Grasgruber et al. [8]. Supplementary Figure S3. Development of the prevalence of overweight and obesity in children (age groups 5, 9, 13, and 17 years) from 1991 to 2021. The classification is based on standardized percentile graphs from the 5th CAV 1991 (90–97th percentile = overweight, ≥ 97th percentile = obese). Sources: 1991: 5th CAV 1991; 1996–2016: The Health of Children; 2021: Study of Anthropological Data of Czech Children (SPLDD) 2021 [9]. Supplementary Figure S4. Relationship between average male and female height in individual districts, according to the place of residence. Supplementary Figure S5. Average percentage of individuals with selected lifestyle characteristics in each quintile of body height. Supplementary Figure S6. Average percentage of individuals with selected lifestyle characteristics in each quintile of body height. Supplementary Figure S7. Average percentage of individuals with selected lifestyle characteristics in each quintile of % body fat. Supplementary Figure S8. Average percentage of individuals with selected lifestyle characteristics in each quintile of % body fat. Supplementary Figure S9. Height of participants in the Czechoslovak Spartakiads (young and middle-aged men) in 1955, 1960, and 1965 (n = 11387) divided regionally by major cities. The average height was 173.7 cm (n = 8168) for the territory of the present-day Czech Republic and 171.3 cm (n = 3128) for present-day Slovakia. Cited in Suchý [26]. Supplementary Figure S10. A) Food supply in the Czech Republic (1948–2023), [file 40101_2025_418_MOESM1_ESM.pdf]

Supplementary material

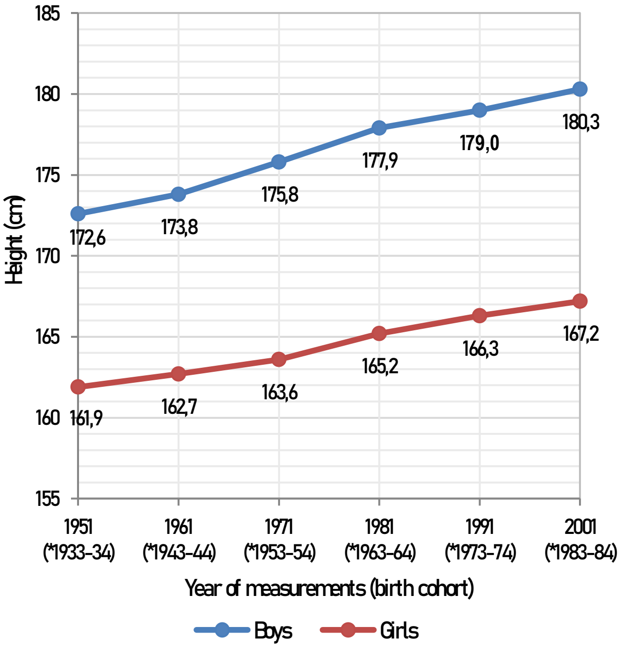

Supplementary Figure 1. Average height of 17-year-old boys and girls in nationwide anthropometric surveys (CAVs) between 1951-2001 (statistically 'smoothed' values). *Source:* Vignerová et al. [4].

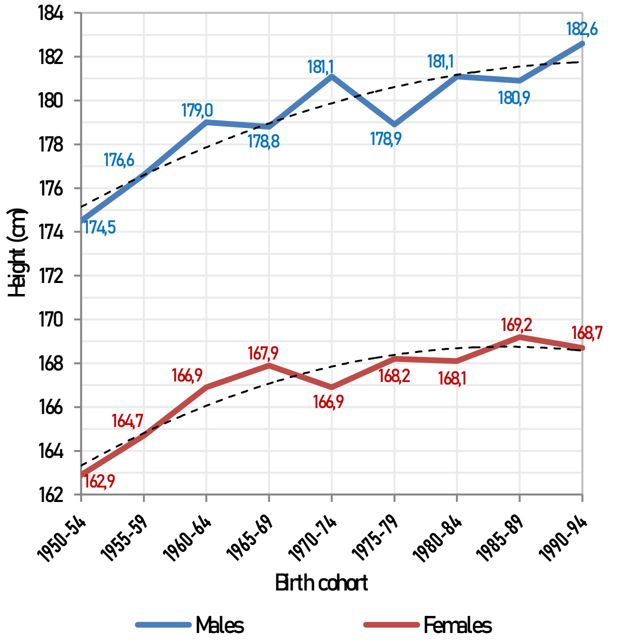

Supplementary Figure 2. Average height of males (n=582) and females (n=656) in the health survey *Physical Fitness in the Czech Republic (2011-2013)*. Note that height in the oldest cohorts is also influenced by the age-related decrease in stature. *Source:* Grasgruber et al. [8].

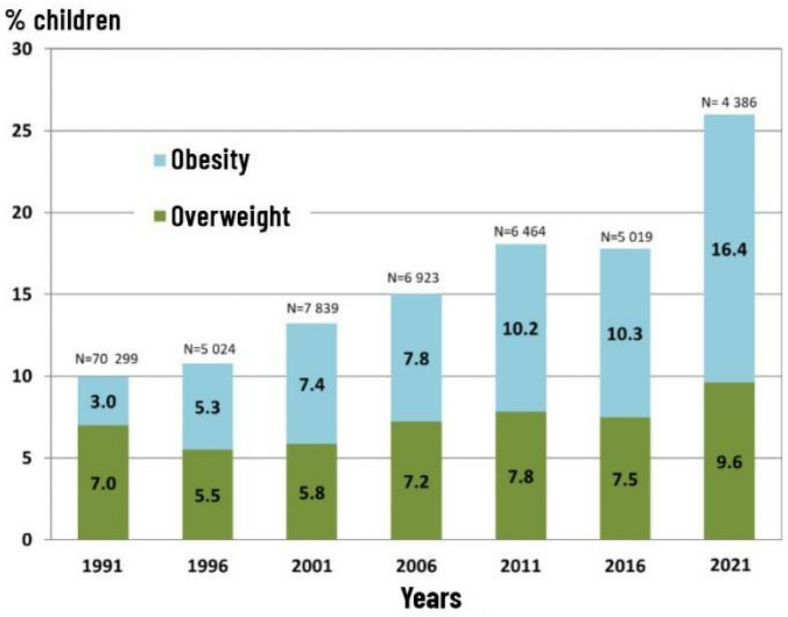

Supplementary Figure 3. Development of the prevalence of overweight and obesity in children (age groups 5, 9, 13, and 17 years) from 1991 to 2021. The classification is based on standardized percentile graphs from the 5th CAV 1991 (90-97th percentile = overweight, ≥ 97th percentile = obese). *Sources:* 1991: 5th CAV 1991; 1996-2016: The Health of Children; 2021: Study of Anthropological Data of Czech Children (SPLDD) 2021 [9].

Supplementary Table 1. Average height in 31 individual schools measured during the first phase of the survey in the Brno-City district.

Schools	School type	Males			Females		
		n	Height (cm)	SD	*n*	Height (cm)	SD
Gymnázium Řečkovice, Terezy Novákové	A	*43*	183.3	6.4	*44*	168.6	6.0
Gymnázium třída kpt. Jaroše	A	*53*	182.3	5.9	*34*	166.3	5.9
Sportovní gymnázium L, Daňka, Botanická	A	*20*	182.0	5.9	*24*	165.5	5.9
Gymnázium Křenová	A	*67*	181.5	6.5	*57*	167.7	6.4
Klasické a Španělské gymnázium, Vejrostova	A	*32*	181.0	7.8	*88*	167.8	6.1
Gymnázium Elgartova	A	*38*	180.9	6.3	*49*	167.5	6.5
Biskupské gymnázium, Barvičova	A	*65*	180.7	7.0	*57*	167.2	5.1
Gymnázium Matyáše Lercha, Žižkova	A	*29*	180.5	5.5	*32*	171.4	5.8
Školský komplex na Mendlově náměstí	A	*37*	180.0	5.3	*26*	165.3	7.6
Gymnázium Vídeňská	A	*74*	180.0	5.7	*57*	166.9	5.8
Gymnázium Hády, Horníkova	A	*4*	178.3	4.4	*2*	171.6	10.0
Obchodní akademie, Střední	B	*10*	186.2	8.9	*33*	166.2	5.4
SOŠ Emila Holuba, Křenová	B	*2*	185.4	1.8	*30*	167.2	5.8
Střední průmyslová škola chemická, Vranovská	B	*52*	182.0	6.2	*55*	167.0	5.1
Střední průmyslová škola, Sokolská	B	*172*	181.0	7.0	*9*	166.0	6.3
Střední průmyslová škola stavební, Kudelova	B	*77*	180.6	7.5	*20*	165.6	4.8
Střední zdravotnická škola, Merhautova	B	*28*	179.1	6.5	*124*	165.7	6.6
Střední škola umění a designu, Husova	B	*14*	178.4	4.9	*28*	167.2	7.3
Bezpečnostně-právní akademie, Rybnická	B	*53*	178.3	5.9	*21*	165.6	6.2
Střední zdravotnická škola, Jaselská	B	*11*	177.2	6.0	*123*	165.0	6.6
SOŠ EDUCAnet, Hudcova	B	*13*	176.8	7.2	-	-	-
SOŠ pedagogická, Lerchova	B	*9*	175.0	4.8	*87*	165.9	6.3
Střední pedagogická škola, Pionýrská	B	-	-	-	*25*	164.3	5.3
SŠ sociálních a zdravotnických služeb Vesna, Údolní	C1	*3*	192.9	1.7	*7*	164.9	9.7
Střední průmyslová škola Purkyňova	C1	*143*	180.7	6.8	*56*	168.0	6.0
SŠ informatiky, poštovnictví a finančnictví, Čichnova	C1	*73*	180.7	7.4	*47*	166.0	6.5
SZŠ Evangelické akademie, Šimáčkova	C1	*4*	175.2	9.3	*39*	166.7	6.3
SŠ potravinářská, obchodu a služeb, Charbulova	C2	*18*	180.8	7.0	*64*	164.3	7.0
Střední škola polytechnická, Jílová	C2	*122*	179.3	6.8	-	-	-
SŠ strojírenská a elektrotechnická, Trnkova	C2	*60*	178.7	6.9	*1*	163.5	
SOU tradičních řemesel, Střední	C2	*12*	178.2	5.0	*33*	166.3	4.9

Supplementary Table 2. Average height in 40 individual schools measured during the second phase of the survey in four regions.

Schools	School type	Males			Females		
		n	Height (cm)	SD	*n*	Height (cm)	SD
Gymnázium Čajkovského Olomouc	A	*32*	182.9	7.6	*44*	166.3	5.7
Gymnázium Kroměříž	A	*12*	182.6	6.9	*24*	166.5	7.6
Gymnázium Tišnov	A	*35*	181.0	6.8	*29*	169.5	4.7
Gymnázium Jihlava	A	*23*	180.9	5.7	*20*	168.5	5.3
Gymnázium Zlín	A	*20*	180.7	5.6	*43*	165.8	6.5
Gymnázium Jiřího Wolkera Prostějov	A	*19*	180.3	9.9	*19*	167.7	5.7
Gymnázium Slovanské náměstí Brno	A	*12*	180.1	4.5	*26*	168.8	5.4
EKO Gymnázium Brno	A	*8*	179.7	8.9	*16*	164.6	5.9
Gymnázium & SOŠ Staré Město (Gymnázium)	A	*10*	179.7	9.2	*16*	170.0	5.2
Gymnázium Židlochovice	A	*13*	179.6	7.3	*7*	168.9	7.6
Gymnázium Dr. Karla Polesného Znojmo	A	*27*	179.2	8.1	*60*	167.6	5.8
Gymnázium Hejčín Olomouc	A	*24*	179.0	6.3	*25*	169.4	7.3
GPOA Znojmo (Gymnázium)	A	*14*	178.8	8.0	*24*	168.5	5.3
Gymnázium Velké Meziříčí	A	*9*	178.8	6.5	*18*	167.4	5.6
Gymnázium T.G. Masaryka Zastávka	A	*31*	178.6	6.6	*23*	164.0	6.2
Gymnázium & SOŠZE Vyškov (Gymnázium)	A	*13*	178.4	4.7	*20*	165.0	5.6
Gymnázium Otrokovice	A	*18*	177.3	6.4	*34*	167.6	6.4
Soukromé gymnázium AD FONTES Jihlava	A	*6*	176.6	9.5	*11*	168.4	8.0
Gymnázium & SOŠZE Vyškov (SOŠZE)	B	*4*	182.5	6.0	*31*	166.4	6.9
SPŠ Přerov	B	*53*	181.9	7.5	*3*	170.1	1.3
GPOA Znojmo (SPŠ & OA)	B	*42*	181.3	6.3	*71*	164.3	5.8
SUPŠ Uherské Hradiště	B	*10*	181.1	5.8	*36*	167.0	6.8
SPŠS Olomouc	B	*80*	180.8	6.8	*4*	166.7	4.5
SZŠ Kroměříž	B	*3*	179.9	10.5	*11*	165.9	5.5
Obchodní akademie Kroměříž	B	*34*	179.3	7.5	*22*	164.9	6.1
SZŠ Znojmo	B	*2*	178.6	6.2	*38*	165.9	5.6
Obchodní akademie a hotelová škola Třebíč	C	*10*	183.3	6.3	*51*	165.8	5.8
SŠ Polytechnická Olomouc	C	*55*	181.0	7.0	-	-	-
SŠ stavební Třebíč	C	*34*	181.0	6.0	*16*	169.6	6.7
Sigmundova střední škola strojírenská Lutín	C	*81*	180.4	7.1	*2*	166.5	4.2
SŠT Přerov	C	*27*	180.2	7.4	*5*	168.3	7.5
SZAŠ Rajhrad	C	*24*	180.1	7.0	*38*	165.1	7.6
SOŠ Prostějov	C	*14*	179.8	6.0	*39*	163.9	9.5
SPŠ OA Uherský Brod	C	*49*	179.8	4.9	*2*	165.2	1.9
Gymnázium & SOŠ Staré Město (SOŠ)	C	*13*	179.7	7.8	*4*	163.8	8.6
SOŠ Jana Tiraye Velká Bíteš	C	*20*	178.7	7.8	*5*	170.7	6.5
SŠHS Kroměříž	C	*10*	178.6	8.9	*44*	167.4	5.9
SOU/SOŠ Znojmo	C	*61*	178.2	6.5	*89*	165.4	6.3
SOU Uherský Brod	C	*25*	177.4	5.7	*15*	164.1	5.0
ISŠ Slavkov u Brna	C	*11*	176.9	7.6	*42*	167.1	7.1

Supplementary Table 3. Average height in 16 individual districts (total sample aged 18-20 years), according to the place of residence.

	Males				Females			
**District**	***n***	**Both parents with university education (%)**	**Height (cm)**	**SD**	***n***	**Both parents with university education (%)**	**Height (cm)**	**SD**
Přerov	*77*	3.9	181.5	7.8	*22*	9.1	166.9	5.4
Jihlava	*30*	10.0	181.0	7.2	*41*	22.0	168.2	6.1
Třebíč	*54*	3.7	180.9	6.2	*91*	5.5	166.3	6.9
Olomouc	*229*	11.4	180.9	6.9	*63*	28.6	168.0	7.0
Brno-Country	*385*	20.0	180.6	6.6	*385*	16.4	166.6	6.2
Brno-City	*787*	26.2	180.6	6.8	*667*	25.8	166.8	6.3
Kroměříž	*67*	16.4	180.0	7.3	*96*	9.4	166.6	6.7
Vyškov	*86*	14.0	179.9	6.7	*151*	10.6	166.5	6.5
Blansko	*81*	12.3	179.7	6.9	*54*	7.4	165.8	5.0
Hodonín	*36*	2.8	179.6	4.5	*51*	0.0	166.0	6.8
Prostějov	*60*	5.0	179.6	7.9	*63*	15.9	165.2	7.3
Zlín	*59*	22.0	179.4	6.2	*98*	19.4	166.6	6.6
Znojmo	*171*	12.3	179.2	6.7	*266*	8.6	166.1	6.2
Uherské Hradiště	*67*	9.0	179.0	5.8	*56*	7.1	166.9	5.9
Břeclav	*44*	9.1	179.0	7.6	*78*	5.1	164.5	5.7
Žďár nad Sázavou	*52*	1.9	178.7	6.8	*53*	13.2	167.3	6.7

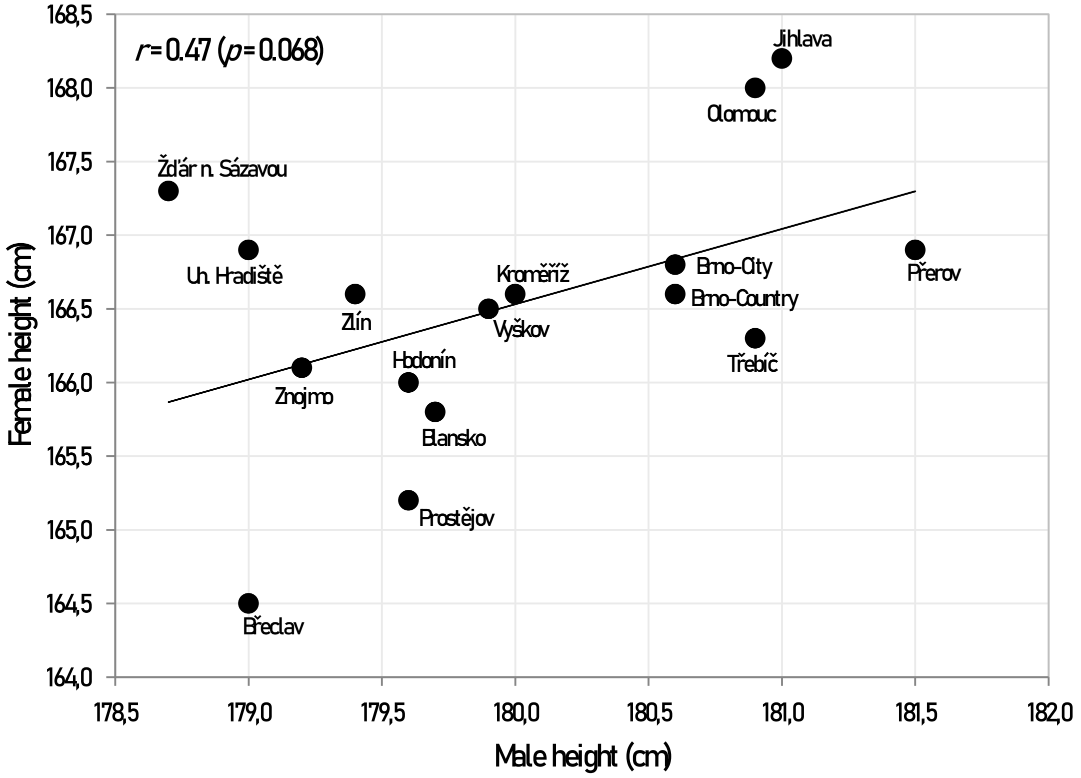

Supplementary Figure 4. Relationship between average male and female height in individual districts, according to the place of residence.

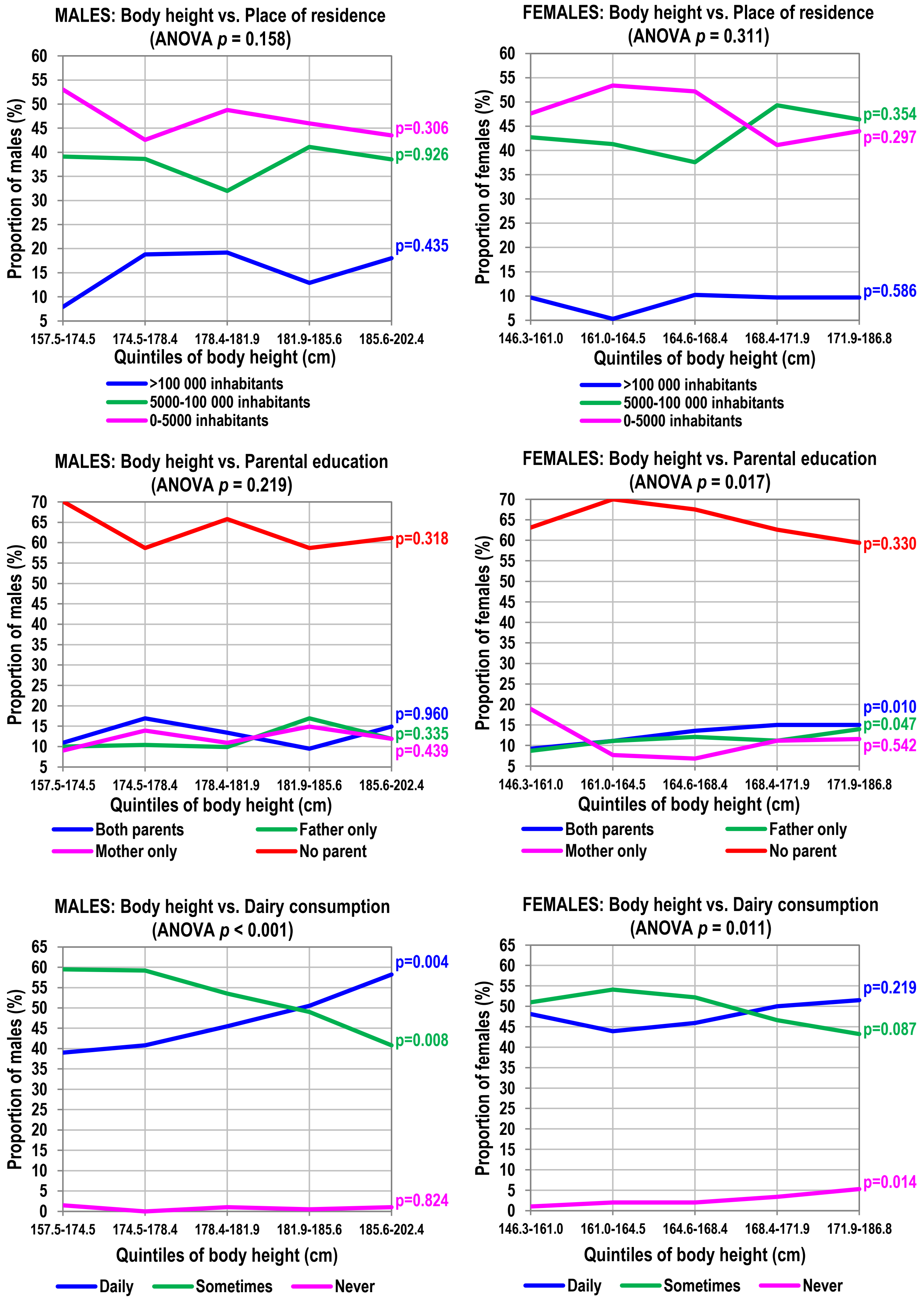

Supplementary Figure 5. Average percentage of individuals with selected lifestyle characteristics in each quintile of body height.

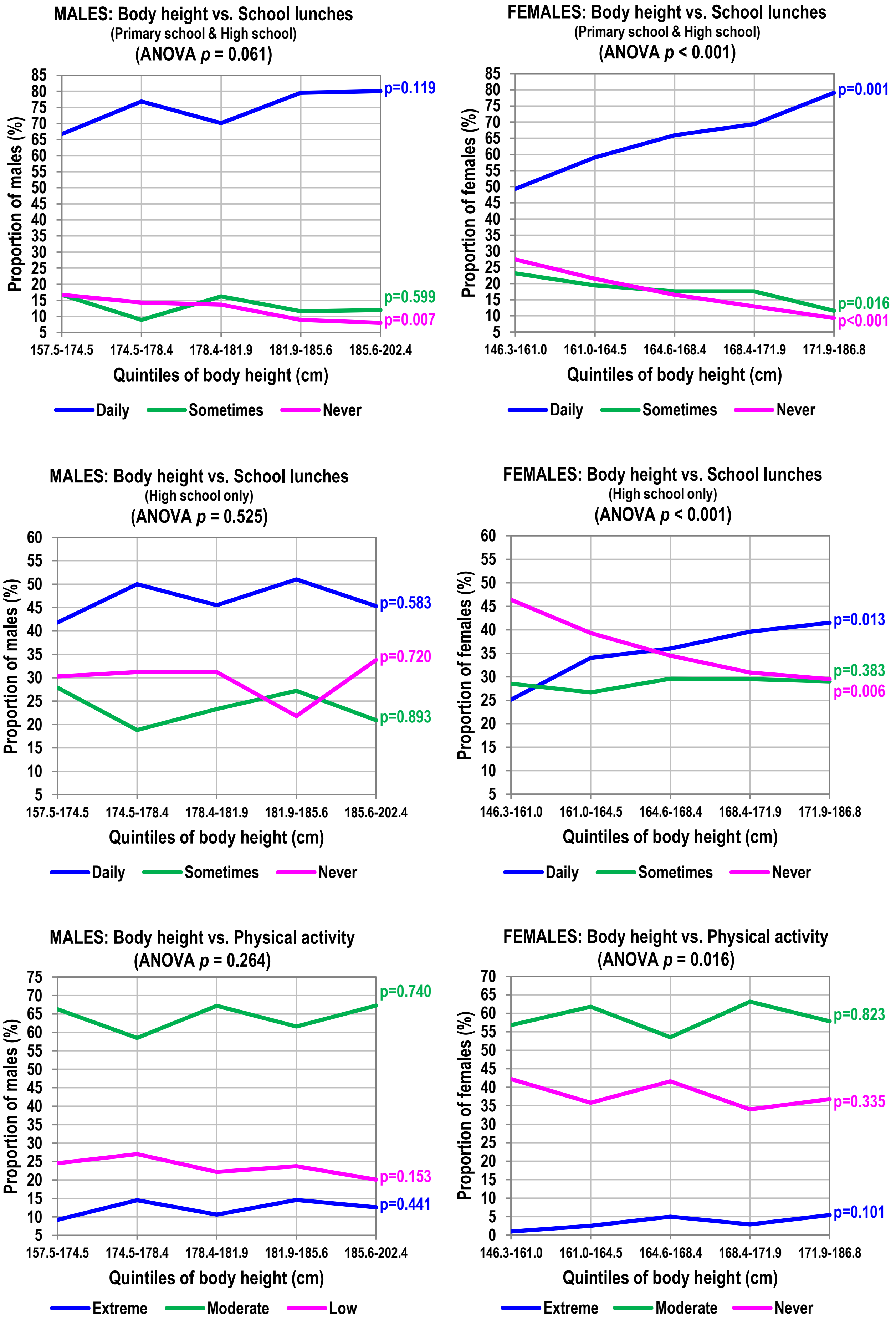

Supplementary Figure 6. Average percentage of individuals with selected lifestyle characteristics in each quintile of body height.

Supplementary Table 4. Average height in various combinations of lifestyle factors.

Parental education	Dairy consumption	School lunches	Males		Females	
			n	Height (cm)	*n*	Height (cm)
BOTH	DAILY		*84*	180.8±7.1	*67*	168.0±6.2
BOTH	SOMETIMES		*48*	180.1±7.1	*59*	166.8±6.5
BOTH		DAILY	*69*	181.6±7.2	*54*	167.5±5.5
BOTH		SOMETIMES	*7*	177.5±7.6	*5*	169.0±4.0
BOTH		NEVER	*3*	177.0±4.3	*4*	162.0±5.5
BOTH	DAILY	DAILY	*43*	181.5±7.8	*26*	167.8±5.5
BOTH	SOMETIMES	DAILY	*26*	181.8±6.3	*27*	166.9±5.5
BOTH	DAILY	SOMETIMES	*3*	183.6±6.4	*3*	166.8±1.5
BOTH	SOMETIMES	SOMETIMES	*4*	172.9±4.9	-	-
BOTH	DAILY	NEVER	*3*	177.0±4.3	*2*	158.0±2.1
BOTH	SOMETIMES	NEVER	-	-	*1*	169.2
	DAILY	DAILY	*206*	181.1±7.3	*134*	168.1±6.5
	DAILY	SOMETIMES	*201*	179.8±6.3	*32*	165.2±5.3
	DAILY	NEVER	*34*	178.4±5.7	*36*	163.3±6.8
	SOMETIMES	DAILY	*201*	179.8±6.3	*133*	167.8±5.7
	SOMETIMES	SOMETIMES	*44*	179.1±6.4	*35*	165.7±5.5
	SOMETIMES	NEVER	*33*	178.6±6.0	*33*	165.3±5.5
NONE	DAILY		*268*	181.2±7.1	*303*	166.5±6.6
NONE	SOMETIMES		*355*	178.7±6.5	*342*	166.1±6.2
NONE		DAILY	*215*	179.5±6.7	*132*	168.7±6.5
NONE		SOMETIMES	*52*	179.1±6.2	*52*	165.2±4.9
NONE		NEVER	*51*	178.5±5.9	*63*	164.6±6.3
NONE	DAILY	DAILY	*99*	180.5±7.0	*65*	168.5±7.3
NONE	SOMETIMES	DAILY	*114*	178.7±6.3	*64*	168.8±5.5
NONE	DAILY	SOMETIMES	*19*	178.1±6.5	*24*	164.6±5.1
NONE	SOMETIMES	SOMETIMES	*32*	180.0±5.9	*26*	165.3±4.7
NONE	DAILY	NEVER	*22*	179.1±5.9	*31*	163.8±7.0
NONE	SOMETIMES	NEVER	*28*	178.1±6.0	*30*	165.2±5.6

Supplementary Table 5. Differences in average height between the extremes of various combinations of lifestyle factors. Significant inter-group differences are highlighted in bold.

Parental education	Dairy consumption	School lunches	Males			Females		
			n	Height (cm)	Difference (cm) (t-test)	*n*	Height (cm)	Difference (cm) (t-test)
BOTH	DAILY		*84*	**180.8±7.1**	***2.1***	*67*	**168.0±6.2**	***1.9***
NONE	SOMETIMES		*355*	**178.7±6.5**	***p=0.008***	*342*	**166.1±6.2**	***p=0.025***
BOTH		DAILY	*69*	**181.6±7.2**	***3.1***	*54*	**167.5±5.5**	***2.9***
NONE		NEVER	*51*	**178.5±5.9**	***p=0.013***	*63*	**164.6±6.3**	***p=0.010***
NONE		DAILY	*215*	179.5±6.7	*1.0*	*132*	**168.7±6.5**	***4.1***
NONE		NEVER	*51*	178.5±5.9	*p=0.353*	*63*	**164.6±6.3**	***p<0.001***
	DAILY	DAILY	*206*	181.1±7.3	*2.5*	*134*	**168.1±6.5**	***2.8***
	SOMETIMES	NEVER	*33*	178.6±6.0	*p=0.063*	*33*	**165.3±5.5**	***p=0.025***
	DAILY	DAILY	*206*	**181.1±7.3**	***2.7***	*134*	**168.1±6.5**	***4.8***
	DAILY	NEVER	*34*	**178.4±5.7**	***p=0.043***	*36*	**163.3±6.8**	***p<0.001***
BOTH	DAILY	DAILY	*43*	181.5±7.8	*1.0*	*26*	167.8±5.5	*0.7*
NONE	DAILY	DAILY	*99*	180.5±7.0	*p=0.451*	*65*	168.5±7.3	*p=0.676*
BOTH	DAILY	DAILY	*43*	181.5±7.8	*3.4*	*26*	167.8±5.5	*2.6*
NONE	SOMETIMES	NEVER	*28*	178.1±6.0	*p=0.051*	*30*	165.2±5.6	*p=0.083*

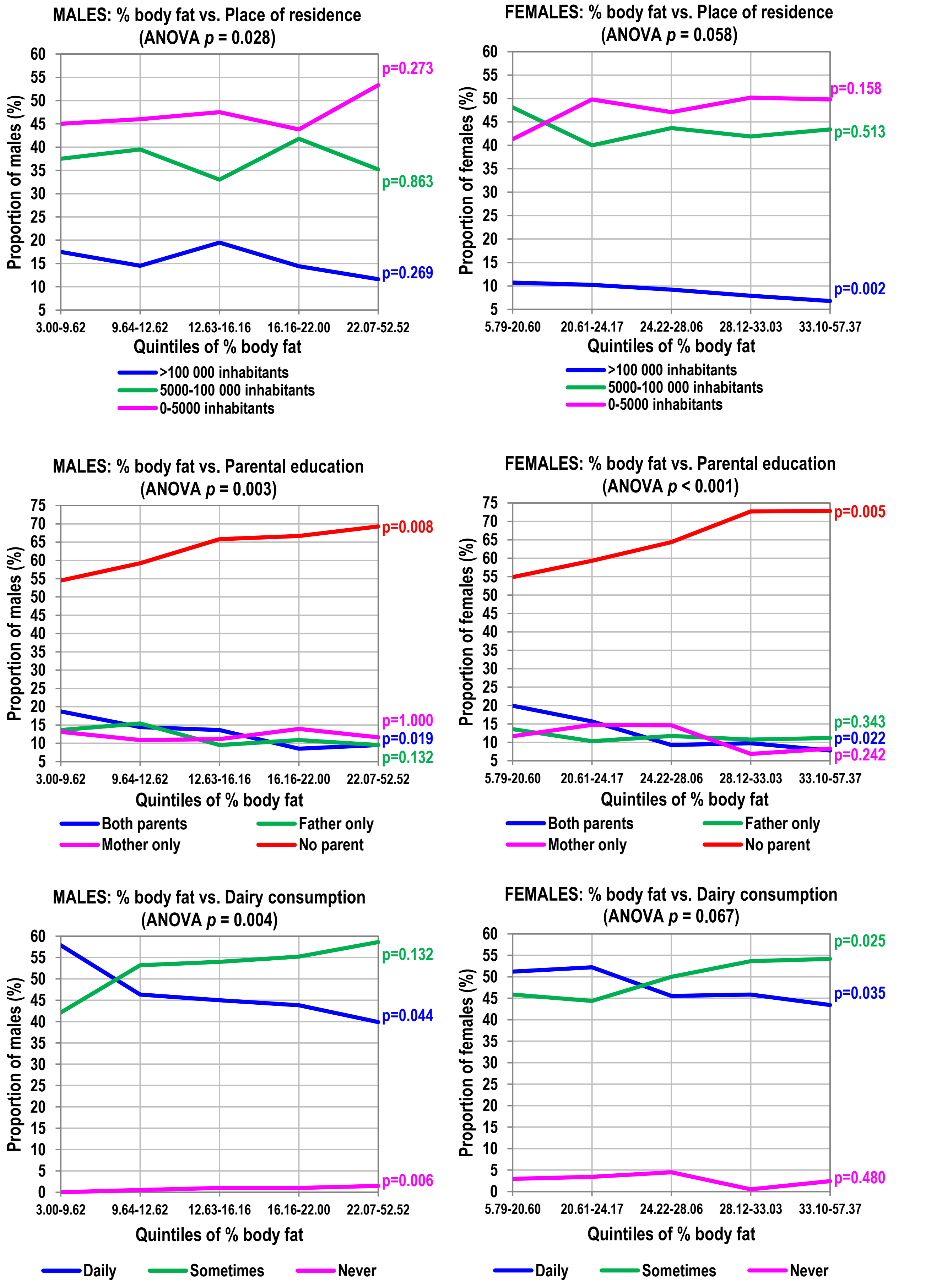

Supplementary Figure 7. Average percentage of individuals with selected lifestyle characteristics in each quintile of % body fat.

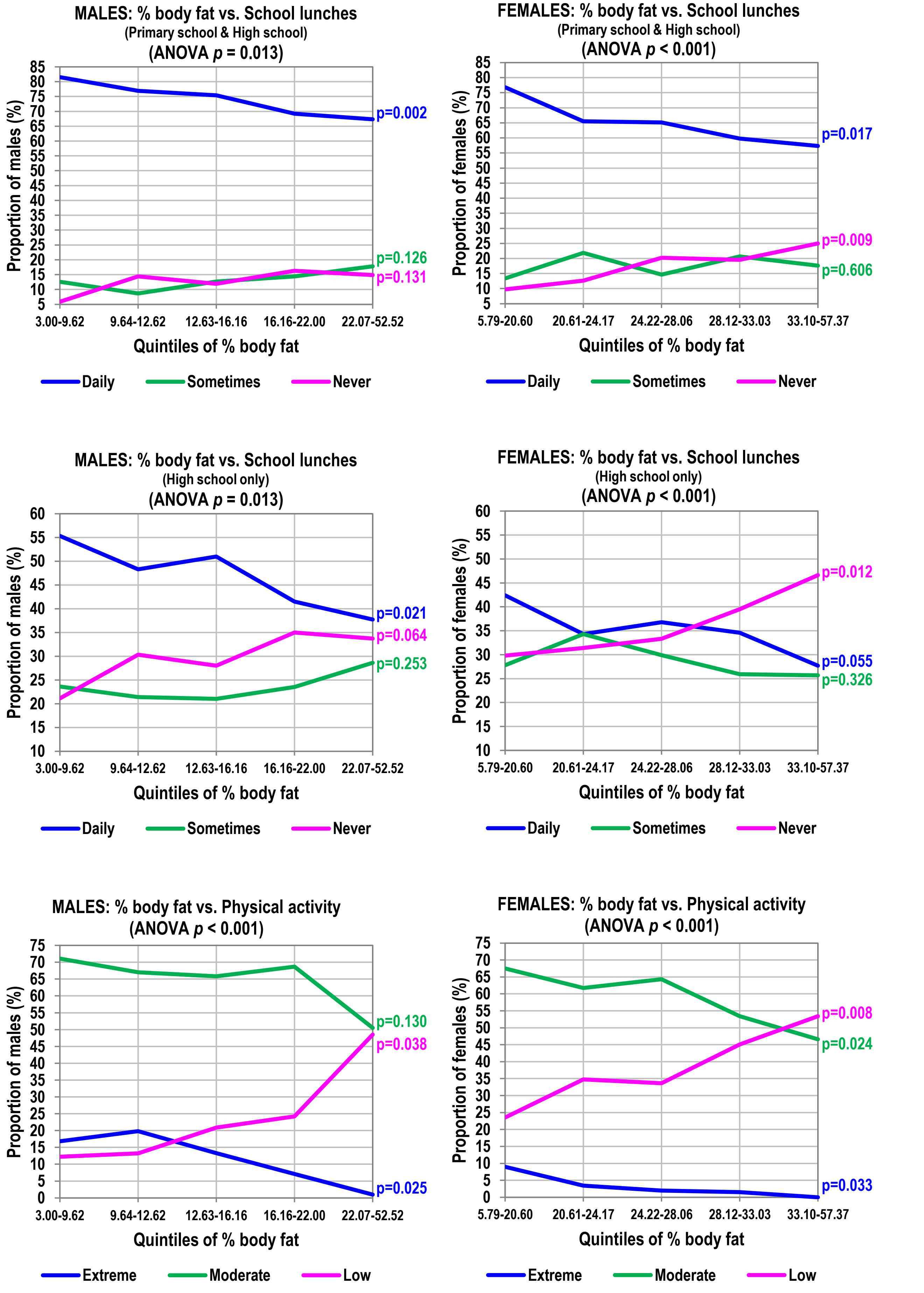

Supplementary Figure 8. Average percentage of individuals with selected lifestyle characteristics in each quintile of % body fat.

Supplementary Table 6. Average % body fat in various combinations of lifestyle factors.

Parental education	Dairy consumption	School lunches	Physical activity	Males		Females	
				n	% body fat	*n*	% body fat
BOTH	DAILY			81	14.36±7.37	64	24.86±6.88
BOTH	SOMETIMES			48	13.59±6.11	58	24.83±6.44
BOTH		DAILY		67	13.85±6.77	51	23.91±6.54
BOTH		SOMETIMES		7	5.65±7.30	4	25.03±4.42
BOTH		NEVER		3	11.33±2.81	4	22.15±3.17
BOTH	DAILY	DAILY		41	14.69±7.22	24	24.39±6.70
BOTH	SOMETIMES	DAILY		26	12.51±5.87	26	23.78±6.44
BOTH	DAILY	SOMETIMES		3	13.47±11.91	2	25.50±6.52
BOTH	SOMETIMES	SOMETIMES		4	17.29±1.96	-	-
BOTH	DAILY	NEVER		3	11.33±2.81	2	20.99±4.93
BOTH	SOMETIMES	NEVER		-	-	1	22.83
	DAILY	DAILY		203	14.59±6.98	132	25.73±6.57
	DAILY	SOMETIMES		27	15.26±7.24	31	25.82±5.95
	DAILY	NEVER		34	16.10±8.93	35	30.05±9.9
	SOMETIMES	DAILY		201	15.55±7.20	132	25.91±6.6
	SOMETIMES	SOMETIMES		44	17.01±7.48	35	28.46±7.44
	SOMETIMES	NEVER		33	19.26±7.69	33	28.58±6.9
	DAILY	DAILY	EXTREME	28	11.46±4.59	6	19.89±5.76
	DAILY	DAILY	MODERATE	132	14.01±6.72	77	24.84±6.61
	DAILY	DAILY	LOW	41	18.58±7.70	48	27.76±5.95
	SOMETIMES	DAILY	EXTREME	21	11.03±3.86	4	21.62±8.38
	SOMETIMES	DAILY	MODERATE	132	15.14±6.05	82	25.09±6.44
	SOMETIMES	DAILY	LOW	45	18.62±9.86	44	28.23±6.00
BOTH	DAILY		EXTREME	13	11.70±5.34	4	18.59±4.40
BOTH	DAILY		MODERATE	49	12.66±4.65	38	24.62±6.38
BOTH	DAILY		LOW	19	20.56±10.55	20	27.20±7.53
BOTH		DAILY	EXTREME	9	11.30±6.58	4	22.29±6.92
BOTH		DAILY	MODERATE	45	12.59±4.57	31	22.75±6.09
BOTH		DAILY	LOW	13	19.96±9.77	15	27.05±6.86
BOTH	DAILY	DAILY	EXTREME	1	14.53	2	20.39±3.35
BOTH	DAILY	DAILY	MODERATE	26	12.81±4.84	12	24.13±6.61
BOTH	DAILY	DAILY	LOW	10	20.63±9.11	10	25.51±7.41
NONE	DAILY			266	15.48±7.49	302	27.45±7.81
NONE	SOMETIMES			354	17.35±7.86	342	28.41±7.66
NONE		DAILY		215	15.48±6.88	132	26.29±6.35
NONE		SOMETIMES		52	16.25±7.41	52	27.81±7.09
NONE		NEVER		51	18.39±9.14	62	30.15±8.32
NONE	DAILY	DAILY		99	14.32±6.62	65	26.29±6.70
NONE	SOMETIMES	DAILY		114	16.54±6.97	64	26.23±6.06
NONE	DAILY	SOMETIMES		19	15.65±7.29	24	25.43±6.06
NONE	SOMETIMES	SOMETIMES		32	16.25±7.40	26	30.18±7.28
NONE	DAILY	NEVER		22	16.19±10.33	30	31.24±9.54
NONE	SOMETIMES	NEVER		28	20.09±8.04	30	28.92±7.04
NONE	DAILY		EXTREME	29	11.24±3.53	10	22.69±4.06
NONE	DAILY		MODERATE	179	14.52±6.85	176	26.51±7.48
NONE	DAILY		LOW	54	21.02±8.42	114	29.31±8.22
NONE	SOMETIMES		EXTREME	27	12.41±4.74	6	14.89±3.51
NONE	SOMETIMES		MODERATE	222	16.46±6.82	185	27.34±6.85
NONE	SOMETIMES		LOW	96	20.34±9.30	146	30.54±7.89
NONE		DAILY	EXTREME	21	10.89±2.86	1	16.71
NONE		DAILY	MODERATE	138	15.01±6.11	84	25.57±6.54
NONE		DAILY	LOW	52	18.36±8.65	45	27.96±5.50
NONE		NEVER	EXTREME	5	12.05±2.79	1	26.81
NONE		NEVER	MODERATE	35	17.56±9.21	34	32.31±8.62
NONE		NEVER	LOW	11	23.92±8.35	25	27.89±7.53
NONE	DAILY	DAILY	EXTREME	11	10.32±2.27	1	16.71
NONE	DAILY	DAILY	MODERATE	66	13.77±6.32	40	25.06±6.97
NONE	DAILY	DAILY	LOW	21	18.01±7.61	23	28.59±5.52
NONE	SOMETIMES	DAILY	EXTREME	10	11.52±3.40	-	-
NONE	SOMETIMES	DAILY	MODERATE	70	16.24±5.70	43	25.82±6.06
NONE	SOMETIMES	DAILY	LOW	31	18.59±9.40	20	27.66±5.65

Supplementary Table 7. Average % body fat in various combinations of lifestyle factors.

Parental education	Dairy consumption	School lunches	Physical activity	Males		Females	
				n	**% body fat**	***n***	**% body fat**
BOTH			MODERATE	*78*	12.80±4.87	*74*	24.04±6.12
NONE			MODERATE	*407*	15.64±6.89	*372*	26.97±7.15
	DAILY		MODERATE	*304*	14.27±6.65	*289*	25.83±7.10
	SOMETIMES		MODERATE	*325*	15.82±6.84	*289*	26.58±7.12
	NEVER		MODERATE	*5*	20.71±4.98	*15*	25.21±6.49
		DAILY	MODERATE	*266*	14.56±6.40	*162*	24.92±6.57
		SOMETIMES	MODERATE	*51*	15.31±6.39	*48*	26.09±6.62
		NEVER	MODERATE	*42*	16.98±8.74	*39*	31.09±8.88
	DAILY	DAILY	MODERATE	*132*	14.01±6.72	*77*	24.84±6.61
	SOMETIMES	SOMETIMES	MODERATE	*33*	15.64±6.99	*24*	27.04±7.34
	SOMETIMES	NEVER	MODERATE	*19*	18.26±7.58	*16*	29.34±7.53
BOTH			LOW	*32*	18.98±9.53	*43*	27.10±6.81
NONE			LOW	*152*	20.65±8.93	*267*	29.92±7.97
	DAILY		LOW	*93*	20.40±8.61	*175*	28.72±7.80
	SOMETIMES		LOW	*138*	19.73±9.22	*201*	29.77±7.70
	NEVER		LOW	*1*	27.95	*11*	26.37±4.86
		DAILY	LOW	*86*	18.60±8.84	*94*	27.89±5.92
		SOMETIMES	LOW	*17*	21.94±8.20	*23*	29.38±6.82
		NEVER	LOW	*17*	22.03±7.72	*28*	27.83±7.64
	DAILY	DAILY	LOW	*41*	18.58±7.70	*48*	27.76±5.95
	SOMETIMES	SOMETIMES	LOW	*10*	22.13±7.32	*11*	31.54±6.98
	SOMETIMES	NEVER	LOW	*11*	22.40±8.05	*15*	27.83±6.42

Supplementary Table 8. Differences in % body fat between the extremes of various combinations of lifestyle factors. Significant inter-group differences are highlighted in bold.

Parental education	Dairy consumption	School lunches	Physical activity	Males			Females		
				n	**% body fat**	**Diff. (% BF) (t-test)**	***n***	**% body fat**	**Diff. (% BF) (t-test)**
BOTH	DAILY			*81*	**14.36±7.37**	***2.99***	*64*	**24.86±6.88**	***3.55***
NONE	SOMETIMES			*354*	**17.35±7.86**	***p=0.002***	*342*	**28.41±7.66**	***p<0.001***
BOTH	DAILY	DAILY		*41*	**14.69±7.22**	***5.40***	*24*	**24.39±6.70**	***4.53***
NONE	SOMETIMES	NEVER		*28*	**20.09±8.04**	***p=0.005***	*30*	**28.92±7.04**	***p=0.020***
BOTH	DAILY		MODERATE	*49*	12.66±4.65	*1.86*	*38*	24.62±6.38	*1.89*
NONE	DAILY		MODERATE	*179*	14.52±6.85	*p=0.075*	*176*	26.51±7.48	*p=0.149*
NONE	DAILY		MODERATE	*179*	**14.52±6.85**	***1.94***	*176*	26.51±7.48	*0.83*
NONE	SOMETIMES		MODERATE	*222*	**16.46±6.82**	***p=0.005***	*185*	27.34±6.85	*p=0.264*
NONE	SOMETIMES		MODERATE	*222*	**16.46±6.82**	***3.88***	*185*	**27.34±6.85**	***3.20***
NONE	SOMETIMES		LOW	*96*	**20.34±9.30**	***p<0.001***	*146*	**30.54±7.89**	***p<0.001***
NONE		DAILY	MODERATE	*138*	15.01±6.11	*2.55*	*84*	**25.57±6.54**	***6.74***
NONE		NEVER	MODERATE	*35*	17.56±9.21	*p=0.051*	*34*	**32.31±8.62**	***p<0.001***
BOTH	DAILY		MODERATE	*49*	**12.66±4.65**	***7.68***	*38*	**24.62±6.38**	***5.92***
NONE	SOMETIMES		LOW	*96*	**20.34±9.30**	***p<0.001***	*146*	**30.54±7.89**	***p<0.001***

Supplementary Table 9. Correlations between body composition and height & body proportions. *R*-values and *p*-values (in parentheses). Significant inter-group differences are highlighted in bold.

**Males (n=348)**	**Body height (cm)**	**Sitting height (cm)**	**Relative sitting height (% body height)**	**Arm span (cm)**	**Relative arm span (% body height)**
	**180.6±7.1**	**95.3±7.6**	**52.80±3.86**	**182.5±9.5**	**101.07±3.76**
**% body fat**	**-0.17 (0.002)**	-0.04 (0.406)	0.04 (0.440)	-0.07 (0.197)	0.08 (0.135)
**Body fat (kg)**	-0.00 (0.957)	0.03 (0.550)	0.04 (0.503)	0.06 (0.300)	0.08 (0.140)
**% muscle mass**	**0.18 (<0.001)**	0.06 (0.253)	-0.03 (0.596)	0.07 (0.177)	-0.09 (0.109)
**Muscle mass (kg)**	**0.61 (<0.001)**	**0.32 (<0.001)**	0.02 (0.659)	**0.46 (<0.001)**	0.00 (0.947)
**Muscle mass index**	0.03 (0.585)	**0.11 (0.033)**	**0.11 (0.044)**	0.07 (0.180)	0.07 (0.184)
**Females (n=215)**	**Body height (cm)**	**Sitting height (cm)**	**Relative sitting height (% body height)**	**Arm span (cm)**	**Relative arm span (% body height)**
	**166.5±6.7**	**89.2±6.0**	**53.63±3.41**	**165.6±9.1**	**99.48±3.86**
**% body fat**	**-0.17 (0.011)**	-0.07 (0.292)	0.03 (0.630)	-0.03 (0.700)	**0.14 (0.035)**
**Body fat (kg)**	0.05 (0.440)	0.04 (0.588)	0.00 (0.944)	**0.15 (0.029)**	**0.16 (0.021)**
**% muscle mass**	**0.23 (0.001)**	0.10 (0.130)	-0.04 (0.601)	0.07 (0.276)	**-0.13 (0.049)**
**Muscle mass (kg)**	**0.71 (<0.001)**	**0.33 (<0.001)**	-0.10 (0.126)	**0.59 (<0.001)**	0.09 (0.211)
**Muscle mass index**	**0.16 (0.016)**	0.12 (0.082)	0.02 (0.772)	**0.22 (0.001)**	**0.15 (0.033)**

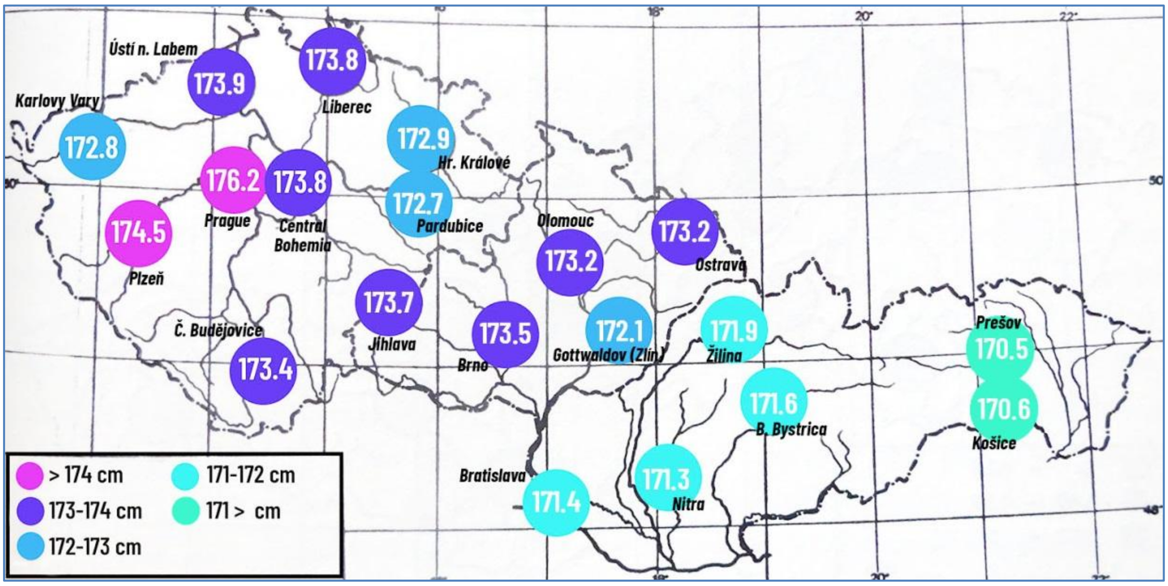

Supplementary Figure 9. Height of participants in the Czechoslovak Spartakiads (young and middle-aged men) in 1955, 1960, and 1965 (*n* = 11387) divided regionally by major cities. The average height was 173.7 cm (*n* = 8168) for the territory of the present-day Czech Republic and 171.3 cm (*n* = 3128) for present-day Slovakia. Cited in Suchý [26].

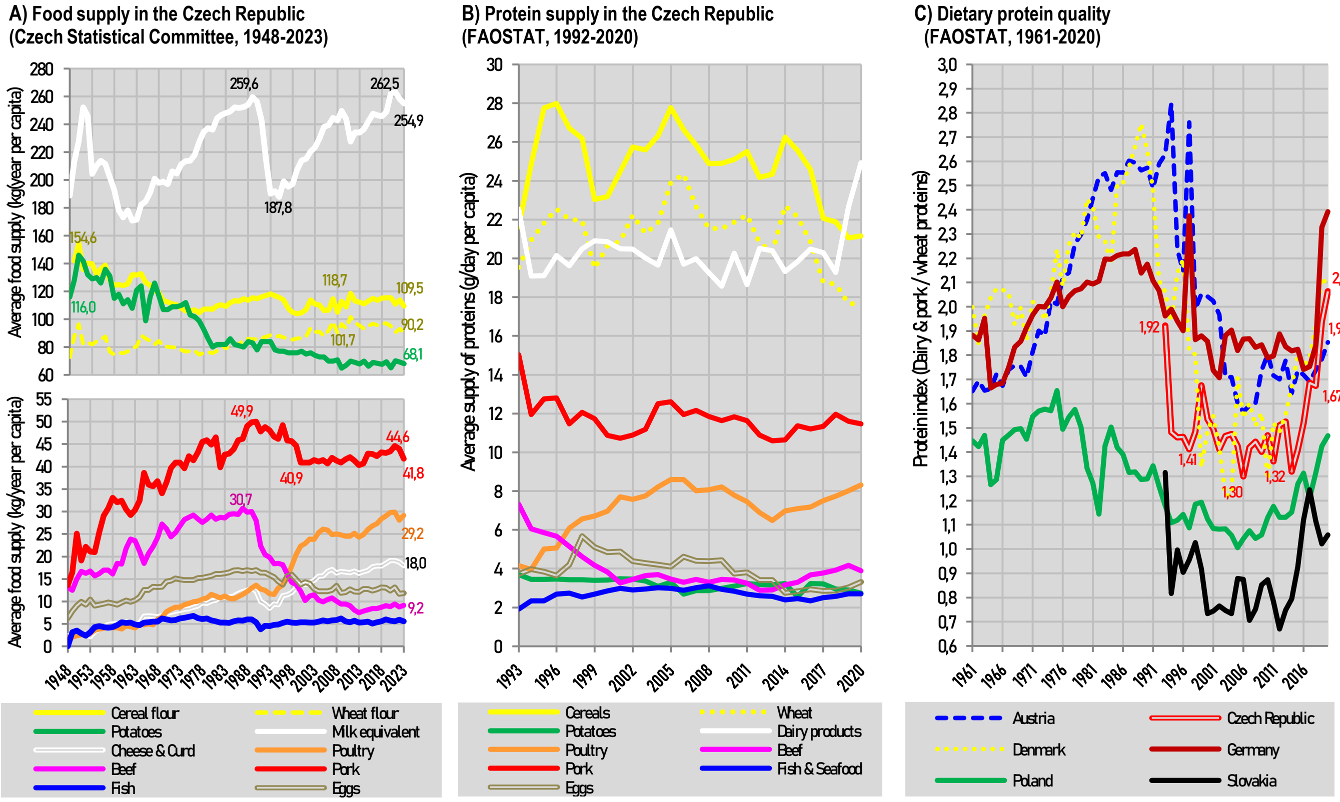

Supplementary Figure 10. A) Food supply in the Czech Republic (1948-2023), according to the Czech Statistical Committee [28]. B) Protein supply in the Czech Republic (1992-2020) according to FAOSTAT [29]. The data precede a revision of the FAOSTAT methodology in 2021. **C) Protein quality (protein index) in the Czech Republic, four neighboring countries, and Denmark (1961-2020), according to FAOSTAT [29].**

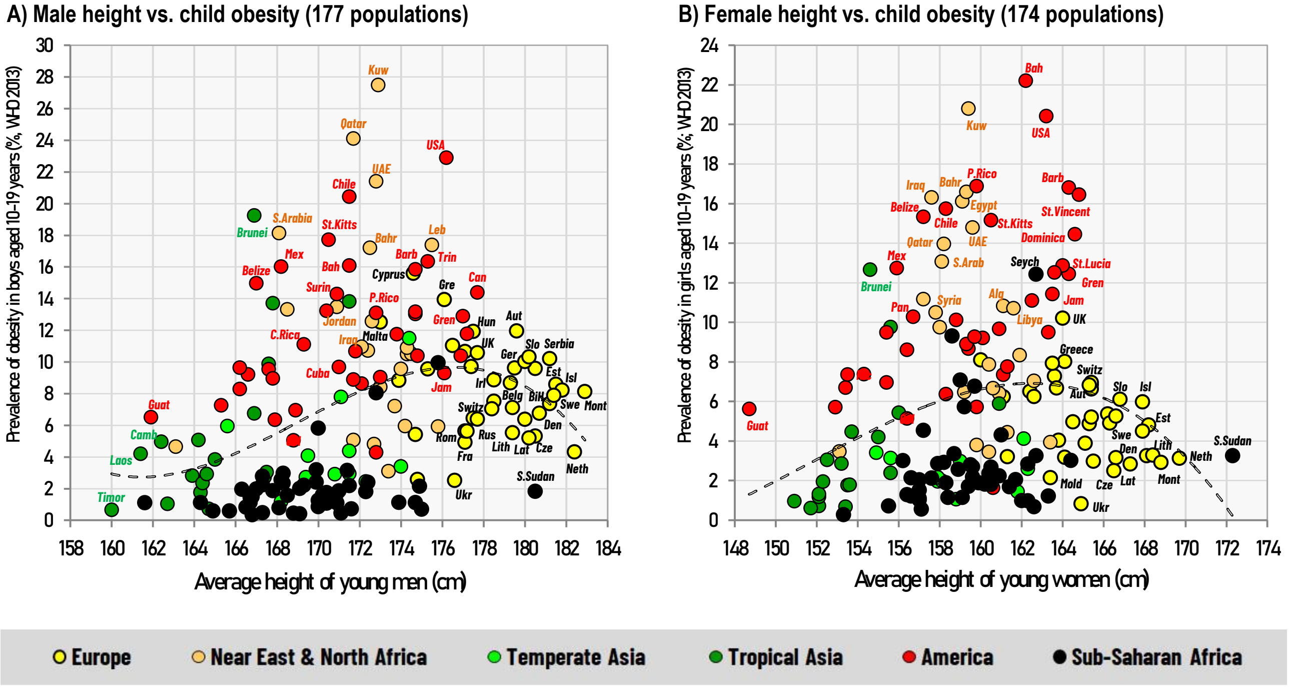

Supplementary Figure 11. A) Relationship between male height and the prevalence of obesity in boys (177 populations). B) Relationship between female height and the prevalence of obesity in girls (174 populations). Data on height come from Grasgruber & Hrazdíra [2] and were supplemented by unpublished data for sub-Saharan Africa. Data on obesity rates in children aged 10-19 years were taken from the WHO website [19]. The graphs do not include Oceania, due to the existence of a highly specific fat-free mass/fat-mass relationship in Oceanian populations.

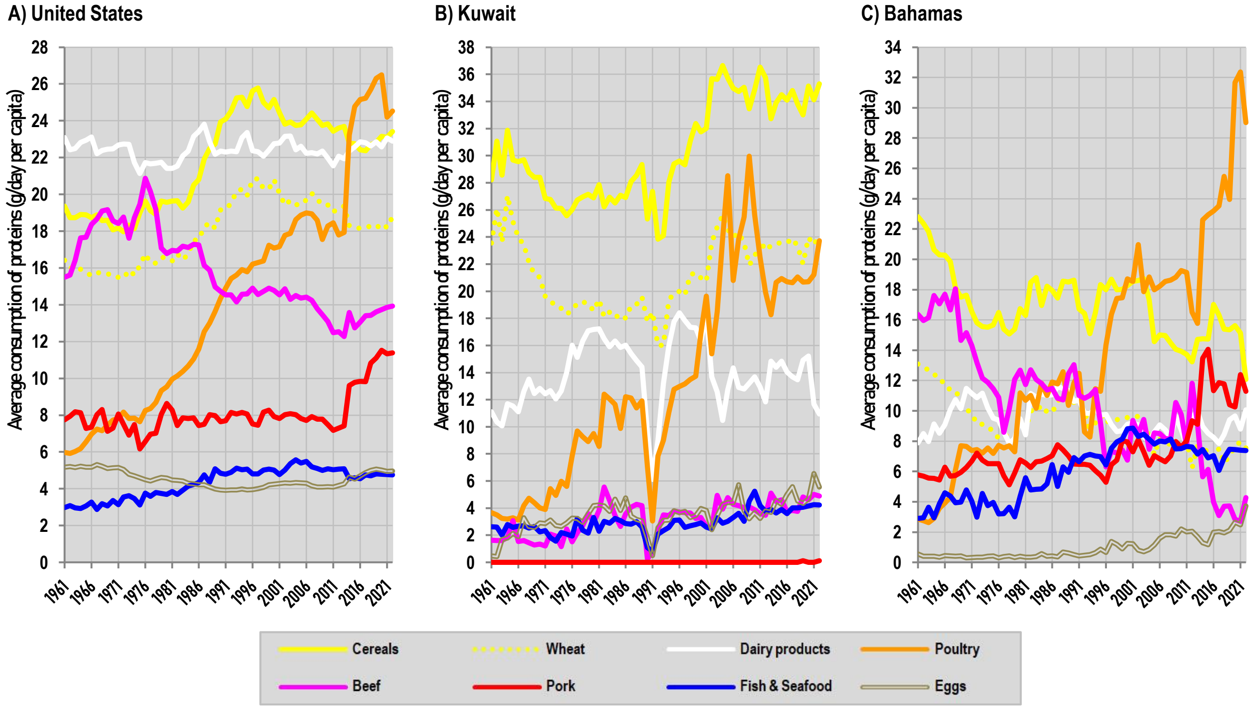

Supplementary Figure 12. A) Trends in protein supply from main food items in the United States between 1961-2022. B) Trends in protein supply from main food items in Kuwait between 1961-2022. C) Trends in protein supply from main food items in the Bahamas between 1961-2022. *Source:* FAOSTAT: Food balances [29]. *Note:* FAOSTAT data for the period 1961-2013 are according to the older FAOSTAT methodology. FAOSTAT data for the period 2014-2022 are according to the new FAOSTAT methodology which was radically updated in 2021. Although the relative trends in consumption development remain the same, the absolute values may differ with the new methodology and are not always comparable with the old methodology.
